# Semisynthetic ‘designer’ p53 sheds light on a phosphorylation–acetylation relay†

**DOI:** 10.1039/d1sc00396h

**Published:** 2021-05-19

**Authors:** Sofia Margiola, Karola Gerecht, Manuel M. Müller

**Affiliations:** Department of Chemistry, King's College London 7 Trinity Street London SE1 1DB UK manuel.muller@kcl.ac.uk

## Abstract

The tumor suppressor protein p53 is a master regulator of cell fate. The activity of p53 is controlled by a plethora of posttranslational modifications (PTMs). However, despite extensive research, the mechanisms of this regulation are still poorly understood due to a paucity of biochemical studies with p53 carrying defined PTMs. Here, we report a protein semi-synthesis approach to access site-specifically modified p53. We synthesized a set of chemically homogeneous full-length p53 carrying one (Ser20ph and Ser15ph) or two (Ser15,20ph) naturally occurring, damage-associated phosphoryl marks. Refolding and biochemical characterization of semisynthetic p53 variants confirmed their structural and functional integrity. Furthermore, we show that phosphorylation within the N-terminal domain directly enhances p300-dependent acetylation approximately twofold, consistent with the role of these marks in p53 activation. Given that the p53 N-terminus is a hotspot for PTMs, we believe that our approach will contribute greatly to a mechanistic understanding of how p53 is controlled by PTMs.

## Abstract 2

‘Designer’ p53: semi-synthesis of the tumor suppressor protein p53 *via* native chemical ligation enables *in vitro* structure–activity studies to reveal how this master regulator of cell fate is itself regulated by phosphorylation.

## Graphical abstract

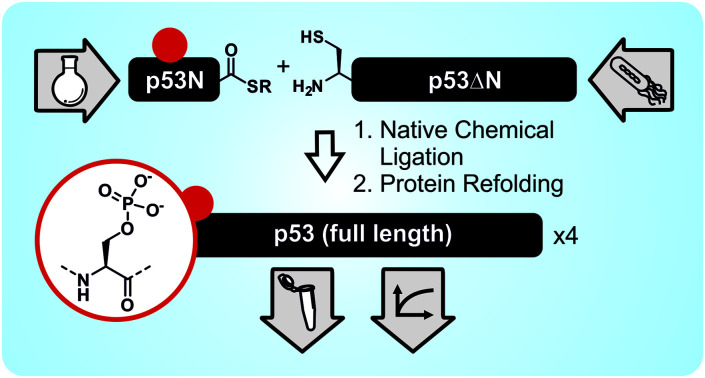

## Introduction

**Fig. 1:**
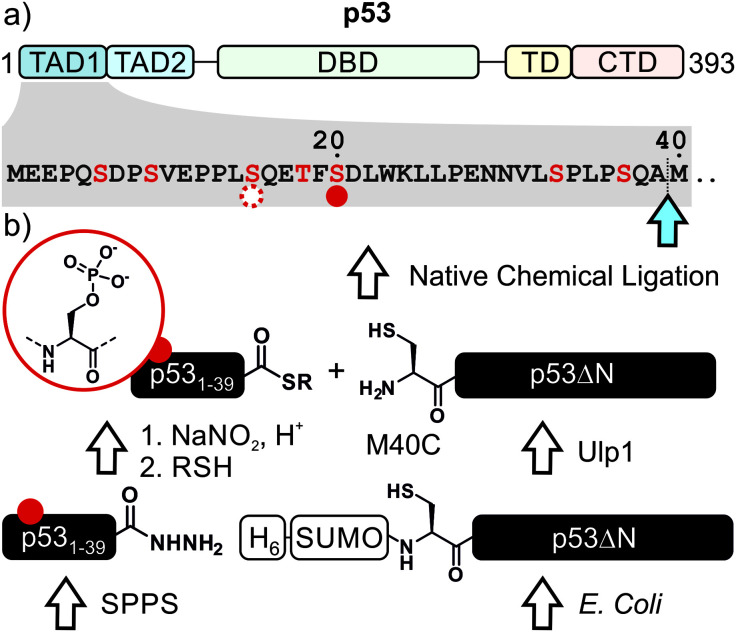
Strategy for synthesis of ‘designer’ p53. (a) Domain architecture of p53. Phosphorylation at Ser20 (S20ph) and Ser15 (S15ph) are indicated with solid and dashed red circles, respectively. Additional known phosphorylation sites are depicted in red. TAD = transcription activation domain; DBD = DNA binding domain; TD = tetramerization domain; CTD = C-terminal domain. (b) Semi-synthesis strategy to access site-specifically phosphorylated p53. The ligation junction is marked with a light blue arrow. To enable native chemical ligation, methionine 40 is mutated to a cysteine (M40C). SPPS = solid-phase peptide synthesis; H_6_ = hexahistidine tag; SUMO = small ubiquitin-like modifier.

p53, often referred to as the guardian of the genome, is a crucial tumor suppressor protein. It orchestrates cell cycle arrest, DNA damage repair and apoptosis in response to cell damage. Given its role in controlling cell fate, it is not surprising that p53 is mutated in many human cancers.^1–3^ p53 acts as a sequence-specific transcription factor. A well-folded DNA-binding domain mediates target-specific recognition of DNA and a neighboring oligomerization domain drives the assembly of active tetramers. These domains are flanked on either side by intrinsically disordered regulatory regions including two N-terminal transactivation domains (TADs; Fig. 1a).^4^ The activity of p53 is tightly controlled by posttranslational modifications (PTMs) such as phosphorylation, acetylation and ubiquitylation, which predominantly occur in the intrinsically disordered regions at the N- and C-termini of p53.^5–7^

Phosphorylation at several sites within the N-terminal TADs are among the first PTMs induced in stress conditions and are associated with p53 activation.^8^ Genetic and peptide-level studies have revealed that these modifications increase p53 stability by impeding a protein–protein interaction with the negative regulator Mdm2.^9,10^ Concomitantly, N-terminal phosphorylation is recognized by transcriptional coactivators such as p300.^11,12^ In turn, p300 acetylates the C-terminal region of p53, which is believed to fine-tune transcriptional activity.^5,13–16^ Thus, many components involved in p53 activation have been described, but the mechanisms of the PTM crosstalks involving the N- and C-terminal regulation hubs remain contentious. To resolve such controversies, quantitative biochemical measurements with site-specifically modified p53 are required.

Previous synthetic biology strategies to prepare chemically defined p53 have yielded invaluable insights into p53 function. For example, regulation of DNA binding by lysine acetylation^17,18^ and PTM cross-talks induced by lysine methylation^19^ have been probed with genetic code expansion technologies.

Moreover, access to segmentally labelled full-length p53 *via* intein-mediated assembly provided new structural insights into molecular recognition by TADs.^20^ Despite these advances, there is a pressing need for new methods to access p53 with customizable PTM states because synthetic biology methods are still limited in the types and numbers of PTMs that can be installed. Chemical synthesis, which represents an ideal route to diversely modified proteins,^21^ has so far been used to prepare only subdomains of p53 (60–100 residue fragments) encompassing the N-terminal^22,23^ or C-terminal^24^ region. Thus, an expansion of these methodologies to obtain full-length p53 with a wider set of compatible PTMs is required to maximize their impact on p53 biochemistry.

Here we report a modular, chemistry driven strategy to synthesize full-length ‘designer’ p53 tetramers. Given the important role that phosphorylation within the first TAD plays in p53 activation,^8,9,25^ we aimed for a strategy that enables flexible installation of PTMs in this region. We synthesized mono- and di-phosphorylated p53 containing phosphoserine residues at positions 15, 20 and a combination thereof; these marks represent initial p53 activation steps. This method allowed us to directly measure downstream signaling events *in vitro*. Overall, this work paves the way for biochemical and biophysical studies on how p53 decides cell fate.

## Results and discussion

### Synthetic strategy for ‘designer’ p53

We decided to harness protein semi-synthesis, a versatile approach to generate large, tailored (phospho-)proteins (Fig. 1b).^26–28^ The method relies on the fusion of synthetic peptides carrying defined chemical modifications to recombinant proteins. These fragments are joined by native chemical ligation, requiring chemically compatible reaction handles, *i.e.* a C-terminal α-thioester and an N-terminal cysteine residue.^29,30^ Because none of the native Cys residues in p53 are located near the N-terminus, we decided to introduce a Cys residue by mutation. Specifically, we chose Ala39 and Met40 as the ligation junction, because (i) the region from 1–39 contains seven critical phosphorylation sites and is expected to be synthetically accessible; (ii) ligations with C-terminal Ala residues proceed readily;^31^ and (iii) Met40 is located between the two TADs and its mutation to Cys is therefore expected to have only a minor effect on TAD function.

### Semi-synthesis of unmodified p53

**Fig. 2:**
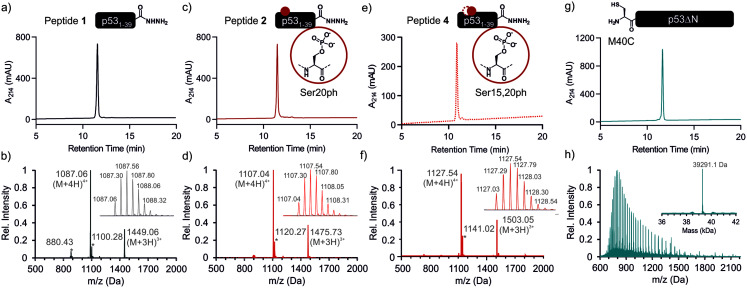
p53 fragments for semi-synthesis. RP-HPLC and MS data for the unmodified peptide **1** ((a and b) expected mass: 4344.18 Da, observed mass: 4344.24 Da), peptide **2** ((c and d) expected mass: 4424.15 Da, observed mass: 4424.16 Da), peptide **4** ((e and f) expected mass: 4424.15 Da, observed mass: 4424.16 Da) and p53ΔN ((g and h) expected mass: 39 292.3 Da, observed mass: 39 291.1 Da). Peaks denoted with an asterisk in the mass spectra are ammonium chloride adducts. The insets depict the isotope distribution of the most intense peak (b, d and f) or the deconvoluted spectrum (h).

We began by synthesizing a p53 peptide encompassing residues 1–39 on solid phase. A C-terminal acyl hydrazide served as a latent α-thioester surrogate.^32^ We functionalized a chlorotrityl-chloride resin with hydrazine in the presence of base,^33^ followed by semi-automated solid-phase peptide synthesis (SPPS) using carbodiimide/Oxyma couplings and N-α-Fmoc-protected amino acids. Met1 was replaced by its isostere norleucine (Nle) to increase stability against oxidation. After purification by reverse phase (RP)-HPLC, the peptide acyl hydrazide was obtained in high purity (peptide **1**, Fig. 2a and b).

To access the C-terminal fragment of p53 encompassing residues 40–393 with an N-terminal Cys (p53ΔN) we opted for a fusion protein strategy (Fig. 1b and S1a†). An N-terminal His_6_-SUMO tag was added, and the construct was produced in *E. coli via* auto-induction,^34^ which provided considerably higher yields (≈80 mg L^−1^) compared to standard IPTG induction (<10 mg L^−1^, Fig. S1b†). The protein was purified from inclusion bodies,^35^ followed by cleavage with the SUMO-protease Ulp1 to expose the N-terminal Cys required for ligation (Fig. S1c†). After purification by reverse nickel affinity chromatography and RP-HPLC, 5–9 mg p53ΔN per L culture were obtained (Fig. 2g and h).

**Fig. 3:**
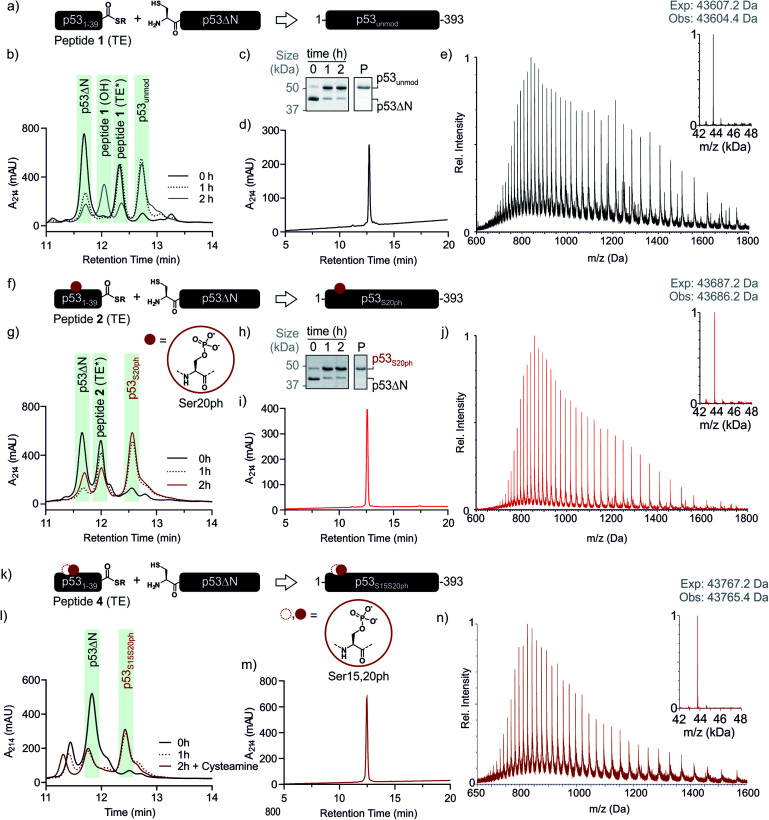
Native chemical ligation of p53 variants. (a) Schematic representation of the ligation reaction to synthesize p53_unmod_. (b and c) Time course of the p53_unmod_ ligation reaction monitored by RP-HPLC and SDS-PAGE, respectively. Mass spectra to identify peptide **1** derivatives (hydrolyzed, OH; thioester (TE*)) are shown in Fig. S3.† Of note, the MPAA-thioester (TE) is converted to a DTT thioester (TE*) prior to analysis. (d and e) RP-HPLC and MS analysis of purified p53_unmod_. The deconvoluted spectrum is shown in the inset (expected mass: 43 607.2 Da, observed mass: 43 604.4 Da). (f) Schematic representation of the ligation reaction to synthesize p53_S20ph_. The S20ph modification is shown as a red circle. (g and h) Time course of the p53_S20ph_ ligation reaction monitored by RP-HPLC and SDS-PAGE, respectively. The hydrolyzed peptide **2** (OH) likely co-elutes with residual p53ΔN. (i and j) RP-HPLC and MS analysis of purified p53_S20ph_. The deconvoluted spectrum is shown in the inset (expected mass: 43 687.2 Da, observed mass: 43 686.2 Da). (k and l) Schematic representation of the ligation reaction to synthesize p53_S15S20ph_ and time course of the p53_S15S20ph_ ligation reaction monitored by RP-HPLC, respectively. (m and n) RP-HPLC and MS analysis of purified p53_S15S20ph_. The deconvoluted spectrum is shown in the inset (expected mass: 43 767.2 Da, observed mass: 43 765.4 Da).

With both fragments in hand, we proceeded to optimizing the ligation reaction. We first tested the conversion of the peptide into an α-thioester (p53_1–39_-SR), the required ligation handle. The acyl hydrazide was oxidized by NaNO_2_ at acidic pH to the corresponding acyl azide, followed by conversion to the corresponding thioester upon addition of 4-mercaptophenylacetic acid (MPAA) at neutral pH (Fig. S2†).^32^ Based on this procedure, we performed a ligation between p53ΔN and 2 eq. *in situ* thioesterified peptide **1** (1.25 mM; 0.25 μmole scale; 10 mg p53ΔN). The reaction was monitored by RP-HPLC and SDS-PAGE upon reduction of reaction aliquots with DTT (Fig. 3a–c). Within 1 h, the peak corresponding to p53ΔN had decreased, with concomitant appearance of a new peak at 12.7 min retention time. Similarly, a gel shift from ∼45 kDa to ∼50 kDa was observed, consistent with ligation to full-length p53. After two hours, we observed >80% conversion to the ligated product. At this time, considerable hydrolysis of the excess peptide thioester occurred and no major increase in product compared to the 1 h time point was observed (Fig. 3b, c and S3†). Upon RP-HPLC purification, we obtained 2.9 mg (≈25% isolated yield) full-length p53_unmod_ (Fig. 3d and e).

### Semi-synthesis of site-specifically phosphorylated p53

Encouraged by these results, we targeted the synthesis of p53 bearing a phosphoserine residue at position 20 (Ser20ph). This modification is naturally installed on p53 by checkpoint kinases in response to DNA damage and is associated with p53 activation.^8,9,25,36^ Peptide **2**, containing the Ser20ph modification, was synthesized by SPPS as described above for the unmodified counterpart **1** and incorporation of phosphoserine with Fmoc-Ser(PO(OBzl)OH)–OH and HATU (Fig. 2c and d; see ESI† for details). The ligation between 0.25 μmoles p53ΔN and 2 eq peptide **2** proceeded rapidly (>85% conversion after 1 h; Fig. 3f–h and S4†). After 2 h, the reaction was quenched and 2.4 mg of full-length p53_S20ph_ were isolated (≈20% yield, Fig. 3i and j).

To install a phospho-group at Ser15, we optimized the SPPS further. Couplings of Ser(PO(OBzl)OH)–OH and subsequent residues were achieved with Oxyma/DIC/DIEA and deprotections with 5% piperazine.^37,38^ These changes improved the overall yield by avoiding guanidinylation and the β-elimination of the phosphoresidues. Thus, peptide **3** bearing the Ser15ph modification was isolated in 9% yield and ligated to p53ΔN (Fig. S5a†). The resulting variant p53_S15ph_ was obtained in 13% yield. Similarly, peptide **4** carrying both the Ser15ph and Ser20ph modifications was synthesised and isolated after two rounds of purification in 3.3% yield (Fig. 2e and f). The ligation between p53ΔN and 2 eq. of *in situ* thioesterified peptide **4** (0.5 μmole scale; 20 mg p53ΔN) was monitored by RP-HPLC (Fig. 3k and l). After 1 h, the reaction was quench by cysteamine to neutralize the unreacted thioesterified peptide and subsequently reduced with DTT. 6.3 mg of full length p53_S15S20ph_ were isolated (≈28% yield, Fig. 3m and n).

We analogously prepared a variant featuring the Ser15ph and Ser20ph marks and an alkyne handle by replacing the N-terminal residue with propargylglycine. This modification would enable derivatization of phospho-p53 with fluorescent labels or affinity tags *via* click chemistry. Ligation on a 0.25 μmole scale yielded 1.7 mg of full-length p53_S15S20ph_ ≡ (16% yield Fig. S5b†).

### Refolding of p53 into biochemically active tetramers

Next, we set out to renature semi-synthetic p53 variants into their native tetrameric state. To optimize the renaturation of p53, we produced untagged full-length p53 in *E. coli* and purified the protein from inclusion bodies (p53_rec_, ≈30 mg L^−1^). We then refolded p53 from 6M GdmCl *via* dilution followed by dialysis based on a procedure adapted from Bell *et al.*^35^ Refolded p53 was concentrated by heparin ion exchange chromatography and purified *via* size exclusion chromatography (SEC). We observed three species eluting at 9, 12–13 (p53-F12), and 14.3 mL (p53-F14), corresponding to soluble aggregates, ∼800 kDa and ∼350 kDa, respectively (Fig. S6a and b†). As expected from previous reports,^39^ p53 elutes at higher molecular weights than expected for the 174 kDa tetrameric complex, presumably due to its unusual structure and flexibility. We turned to chemical crosslinking with glutaraldehyde to assess the oligomeric state of the fractions. When adding increasing amounts of glutaraldehyde, p53-F14 initially formed covalent dimers, followed by a predominantly tetrameric product, suggesting that this fraction represents well-folded p53 (Fig. S6c†). By contrast, p53-F12 only crosslinked into higher molecular weight oligomers.

**Fig. 4:**
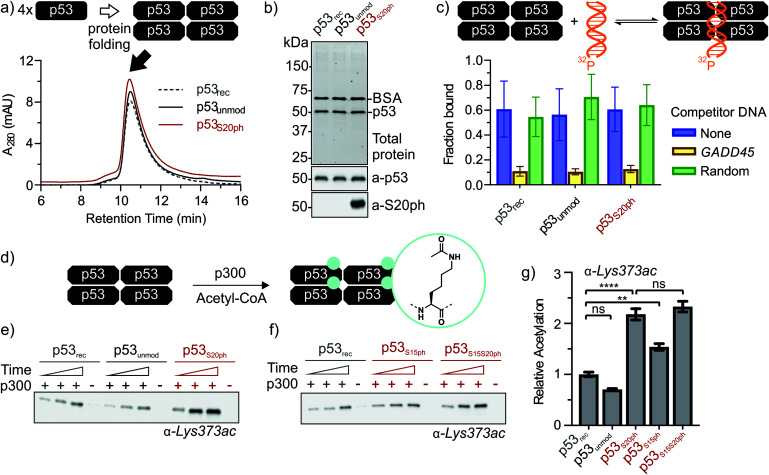
Functional characterization of ‘designer p53 tetramers’. (a) Analytical size exclusion chromatograms of p53 variants. (b) SDS-PAGE and western blot of p53 variants. 0.1 mg mL^−1^ BSA was added to samples prior to analysis. Lanes 1, 2, and 3 correspond to p53_rec_, p53_unmod_ and p53_S20ph_, respectively. Top: total protein was visualized *via* in-gel stain-free detection. Middle: p53 was detected with antibody pAb240. Bottom: the S20ph modification was detected with antibody ab157454. (c) p53 variants bind to DNA site-specifically. The fraction of p53-bound DNA was determined *via* electrophoretic mobility shift assay with radiolabeled target DNA (*GADD45*, blue). Target specificity was evaluated by the addition of excess unlabeled target (yellow) or sequence-randomized (green) DNA probes. (d) Reaction scheme for the p300-catalyzed acetylation of C-terminal lysine residues of p53. (e and f) p300 acetyltransferase assays with p53 variants as substrates. Western blots were probed with a site-specific α-Lys373ac antibody. Time points are 2, 5, and 10 min. Negative controls in the absence of p300 were incubated for 10 min. Relative rates for Lys373 acetylation are plotted in (g). Error bars depict the error of the fit from 3 independent measurements for p53_rec_, p53_unmod_ and p53_S20ph_ and 4 independent measurements for p53_S15ph_ and p53_S15S20ph_. One-way ANOVA with Turkey's multiple comparison was used to test the statistical significance. *P*-values obtained for p53_rec_*vs.* p53_unmod_: 0.182, for p53_rec_*vs.* p53_S20ph_: <0.0001, for p53_rec_*vs.* p53_S15ph_: 0.0039, p53_S20ph_*vs.* p53_S15S20ph_: 0.693.

We repeated the refolding procedure for semi-synthetic p53_unmod_ and p53_S20ph_ and isolated the 14 mL SEC fractions. All variants refolded reproducibly into tetrameric species (>90% purity by analytical SEC) and yielded sufficient material for functional studies (Fig. 4a and S6d†). SDS-PAGE and western blotting confirmed the identity and purity of p53 preparations, as well as the presence of phosphoserine in p53_S20ph_ (Fig. 4b). To further validate the functionality of refolded p53, we tested whether the variants bind specifically to p53 target DNA (*GADD45*). Indeed, electrophoretic mobility shift assays^40,41^ demonstrated that our p53 variants bound to radiolabeled *GADD45* targets (Fig. 4c and S7†). For all variants, this binding could be competed away with excess unlabeled *GADD45* DNA, but not with a sequence scrambled competitor. Collectively, these observations confirm that refolded p53 variants are tetrameric and active *in vitro* as site-specific DNA binding proteins.

### p53 phosphorylation enhances downstream acetylation

Genetic and peptide-level studies have shown that p53 phosphorylation activates p53 *via* the acetyltransferase p300.^5,11–16,42,43^ Specifically, interaction studies with p53 phospho-peptides and isolated p300 domains have shown that phosphorylation increases binding of p53 to several p300 domains.^44–47^ However, it is not known whether increased p300 binding to the p53 N-terminus indeed stimulates acetylation of distal lysines in the p53 C-terminus. To answer this question, we incubated tetrameric p53_rec_, p53_unmod_ and p53_S20ph_ with p300 in the presence of acetyl-CoA and measured p53 acetylation by western blot (Fig. 4d, e and S8†). Notably, the presence of a phosphoserine residue in p53_S20ph_ boosts p300-dependent acetylation of p53 by 2.2-fold. This enhancement is seen both for site-specific acetylation at Lys373 (Fig. 4e and g), one of the main acetylation sites,^13^ and when probing for global acetylation (Fig. S8e–g†).

To gain further insight into this crosstalk, we also refolded semisynthetic p53_S15ph_ and p53_S15S20ph_ and subjected these variants to p300 assays. Single phosphorylation at Ser15 enhanced acetylation by about 1.5-fold, thus showing a distinct response compared to p53_S20ph_. Combination of both PTMs did not result in a further increase above the enhancement provided by phosphorylation at Ser20 (2.3 ± 0.2 *vs.* 2.2 ± 0.2).

Thus, enhanced binding of phospho-p53, observed previously at the level of peptides,^46,47^ translates into increased acetyltransferase activity on full-length tetramers. This result is consistent with the biological model where p53 phosphorylation precedes p300-dependent acetylation, which ultimately leads to transcriptional activation.^42^

**Fig. 5:**
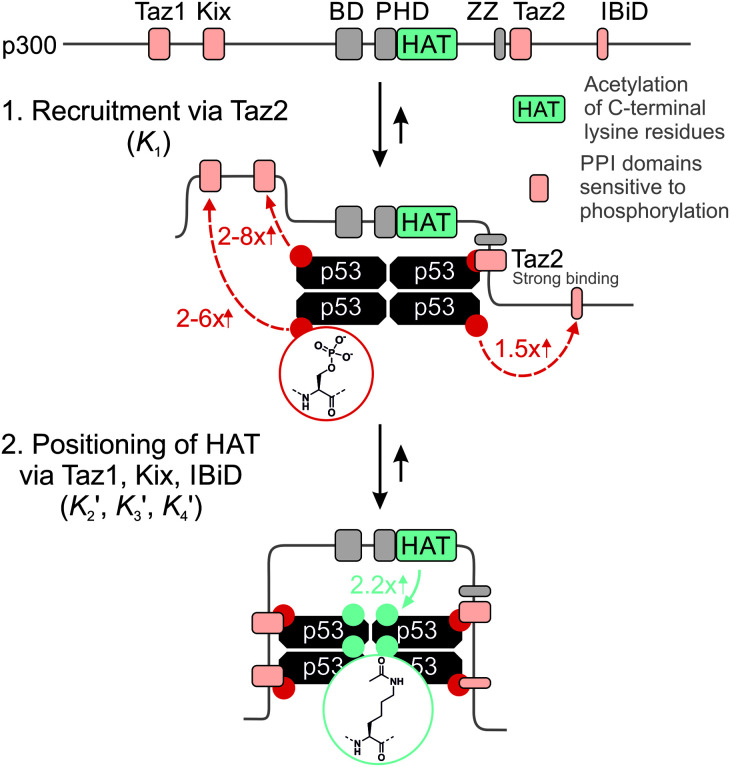
Model for phospho-p53 acetylation by p300. p300 is recruited to p53 by Taz2 binding to (phospho)-p53 and followed by intramolecular engagement of Taz1, Kix and IBiD. Red arrows indicate the relative enhancement of peptide binding by the S20ph modification to the relevant domains determined by Teufel *et al.*^46^ and Lee *et al.*^47^ The teal arrow indicates enhanced acetylation of p53_S20ph_ by p300.

The 1.5- to 2.3-fold stimulation of p300 activity by phosphorylation at Ser 15 and/or Ser20 is similar in magnitude to changes induced by p53 peptide phosphorylation for binding to a single p300 domain (1.1-8-fold increase).^46,47^ Initially we were surprised by this observation because p300 is thought to engage p53 *via* four domains (Taz1, Kix, Taz2, IBiD; Fig. 5).^48^ Binding to each of these domains is strengthened by phosphorylation (in the case of Ser20ph, binding is increased by 2-6x for Taz1, 2-8x for Kix, 4x for Taz2, 1.5x for IBiD).^46,47^ Assuming that the ΔΔ*G* values for phosphorylation of each of the four copies of p53 to individual domains are additive, a 50-100-fold increase in the overall binding affinity of phospho-p53 to p300 would be expected.^46^ However, if one assumes that for optimal catalysis, all four domains need to be engaged, this factor is attenuated. Notably, this scenario is consistent with observations that oligomerization-deficient p53 variants show impaired C-terminal acetylation.^49^

A simplified model based on sequential binding of each subunit of p53 to p300 is illustrative:
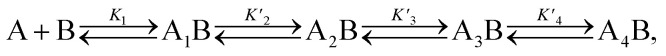
where A and B are the p53 tetramer and p300, respectively, and indices represent the number of p53 subunits engaged in the complex. In this model, *K*_1_ represents the intermolecular dissociation constant of the p300 domain which binds p53 most strongly. All subsequent binding steps are intramolecular and thus described by unit-less dissociation constants 
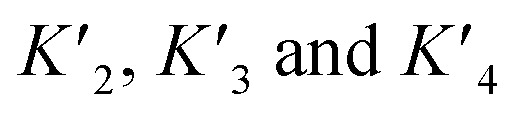
. Their absolute values are unknown.^50^

The p300 Taz2 domain interacts with p53 N-terminal peptides most tightly (K_d_ ∼20 nM)^48^ and exhibits an extremely rapid association rate of >10^10^ M^−1^ s^−1^;^51^ we thus assume that this interaction occurs first (*K*_1_). Under the conditions of our assay (300 nM p53), it is likely that the Taz2 domain is fully bound, regardless of phosphorylation state. For simplicity, we assign the next binding event – based on peptide *K*_d_ values – to Taz1 (*K*_d_ ∼1 μM), followed by Kix (*K*_d_ ∼3 μM) and IBiD (*K*_d_ ∼8 μM).^48^ Using the steady-state assumption, the fraction of p300 with all sites bound under saturating concentrations of p53 ([A] ≫ *K*_1_) can be approximated as:
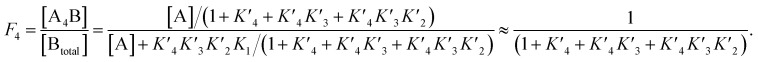
The effect of p53 phosphorylation on tetravalent binding of p300 can therefore be defined as:
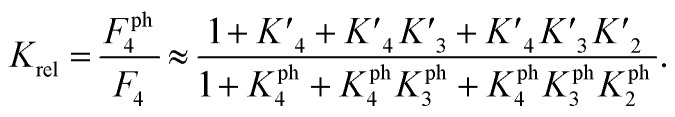
To estimate *K*_rel_ for p53_S20ph_, we approximate 
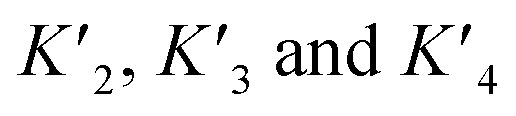
 based on the average intermolecular binding affinities from Teufel *et al.*^46^ and Lee *et al.*^47^ and arbitrarily chosen effective molarities provided by the initial engagement of p300 and p53. At an effective molarity of 0.3, 3 or 30 μM, *K*_rel_ is 14, 2.1 or 1.1. In these respective cases, approximately 2%, 45% or 91% of p300 are tetravalently bound to p53_S20ph_. Consistent with our data, this model predicts that at 3 μM effective molarity, the *K*_rel_ values for p53_S15ph_ and p53_S1520ph_ are 1.7 and 2.6, respectively.

Our results are thus fully compatible with previous estimates from peptide binding studies and corroborate that neither single nor double-site phosphorylation produces an all-or-none-response. Instead, our results support a more modest effect of S15 and/or S20 phosphorylation, which can be explained by a requirement for simultaneous engagement of p300 by all four N-terminal tails of the p53 tetramer to promote efficient acetylation. This scenario would open the opportunity for a more graded response depending on the nature and degree of stress by tunable activation upon phosphorylation at distinct binding interfaces.

## Conclusions

p53 plays a key role in defending against tumor formation. Accordingly, it is among the most studied proteins to date. However, difficulties in obtaining defined forms of p53 have hampered a mechanistic understanding of how this protein controls cell fate decisions. Isolation from various recombinant sources including *E. coli*, baculovirus-infected insect cells and mammalian cells provides p53 with different biochemical properties.^52,53^ To bypass issues of protein heterogeneity, synthetic peptides are frequently used as proxies for full-length proteins. Measurements with peptides derived from intrinsically disordered regions of p53 have yielded quantitative insights into the role of PTMs in controlling protein–protein interactions. However, because p53 is a multi-domain protein, which is active as a tetramer, peptide-based studies cannot be used to address more complex biochemical and biophysical phenomena. To overcome this limitation, we have developed a protein semi-synthesis strategy to generate chemically defined p53 tetramers. Using a combination of chemical synthesis of N-terminal phospho-peptides, recombinant production of truncated p53 and native chemical ligation we accessed milligram quantities of pure, site-specifically mono- and di-phosphorylated p53.

Based on ‘designer p53’, we have explored an interplay of PTMs located ∼350 amino acids apart: phosphorylation of Ser residues in the N-terminus, and acetylation of Lys residues in the DNA-binding domain and the C-terminus.^5^ Our results support a crosstalk between phosphorylation of the p53 N-terminus and downstream acetylation, where individual phosphoryl marks result in subtle yet distinct enhancement of p300 activity. This mechanism as well as other biochemical processes controlled by p53 PTMs can now be fully explored based on ‘designer’-p53 substrates such as the ones described herein. Moreover, given the modularity of protein semi-synthesis,^21^ we anticipate that our approach is readily extended to diverse PTMs and their combinations, and thus paves the way for biochemical studies on how p53 contributes to tumor prevention.

## Funding sources

This work was supported by the Wellcome Trust and the Royal Society (Sir Henry Dale Fellowship 202250/Z/16/Z to MMM), King's College London (Studentship to SM) and the London Interdisciplinary Doctoral Programme (Studentship to KG).

## Data availability

The data that support the findings of this study are available from the corresponding author, MMM, upon reasonable request.

## Author contributions

SM, KG, MMM designed, performed and analysed the experiments and wrote the manuscript; MMM conceived the project and supervised the work.

## Conflicts of interest

There are no conflicts to declare.

## Supplementary Material

SC-012-D1SC00396H-s001
