# Supplementary material for: Semisynthetic ‘designer’ p53 sheds light on a phosphorylation–acetylation relay
Source: Chem Sci. 2021 May 19;12(24):8563–70. doi: 10.1039/d1sc00396h (PMC8221199; doi:10.1039/d1sc00396h)
Supplement: SC-012-D1SC00396H-s001 [file SC-012-D1SC00396H-s001.pdf]

Electronic Supplementary Material (ESI) for Chemical Science.
This journal is © The Royal Society of Chemistry 2021

Supporting Information for:

Semisynthetic ‘designer’ p53 sheds light on a phosphorylation-acetylation relay

Sofia Margiola‡, Karola Gerecht‡, Manuel M. Müller*

King’s College London, Department of Chemistry, 7 Trinity Street, London SE1 1DB, United Kingdom

‡SM and KG contributed equally to this work.
*To whom correspondence should be addressed; email: manuel.muller@kcl.ac.uk

Table of Contents

Supporting Figures 4
Fig. S1. Production and purification of p53ΔN. 4
Fig. S2. Optimization of peptide thioesterification. 5
Fig. S3. Identification of reaction intermediates in the ligation of $p53_{unmod}$. 6
Fig. S4. Identification of reaction intermediates in the ligation of $p53_{S20ph}$. 7
Fig. S5. Native chemical ligation of $p53_{S15ph}$ and $p53_{S15S20ph}$-≡. 8
Fig. S6. Analysis of p53 oligomeric state. 9
Fig. S7. Refolded p53 binds DNA sequence-specifically. 10
Fig. S8. p53 acetylation assays. 11
Experimental Procedures 12
Materials and Methods 12
General Procedure for Reverse-Phase Chromatography 12
Mass Spectrometry 12
General Procedure for Peptide Synthesis 1 13
General Procedure for Peptide Synthesis 2 13
Hydrazine functionalization of resin 13
Synthesis of p53(1-39)-$NHNH_2$ 14
Fig. S9. Analysis of p53(1-39)-$NHNH_2$. 15
Synthesis of peptide **1** 16
Fig. S10. Analysis of purified peptide 1. 17
Synthesis of peptide **2** 18
Fig. S11. Analysis of purified peptide 2. 19
Synthesis of peptide **3** 20
Fig. S12. Analysis of purified peptide 3. 21
Synthesis of peptide **4** 22
Fig. S13. Analysis of purified peptide 4. 23
Synthesis of peptide **5** 24
Fig. S14. Analysis of purified peptide 5. 25
Synthesis of $p53_{369-383}$ for EMSAs 26
Fig. S15. Analytics of p53369-383 peptide. 26
Production and purification of $p53_{rec}$ 26
Fig. S16. Analytics of $p53_{rec}$. 27
Construction of His-SUMO-p53ΔN. 28
Production of p53ΔN 28
General Procedure for Native Chemical Ligation 1 28
General Procedure for Native Chemical Ligation 2 28
General Procedure for Native Chemical Ligation 3 29
Semi-synthesis of $p53_{unmod}$ 29
Fig. S17. Analytics of purified $p53_{unmod}$. 30
Fig. S18. Analytics of purified $p53_{unmod}$. 31
Semi-synthesis of $p53_{S20ph}$ 32

Fig. S19. Analytics of purified p53$_{S20ph}$. 33
Semi-synthesis of p53$_{S15ph}$ 34
Fig. S20. Analytics of purified p53$_{S15ph}$. 35
Semi-synthesis of p53$_{S15S20ph}$ 35
Fig. S21. Analytics of purified p53$_{S15S20ph}$. 36
Semi-synthesis of p53$_{S15S20ph}$-≡ 37
Fig. S22. Analytics of purified p53$_{S15S20ph}$-≡. 38
Refolding of p53 38
Characterization of Oligomeric State 38
DNA Binding Assays 38
Acetyltransferase assays 39
SI References 40

Supporting Figures

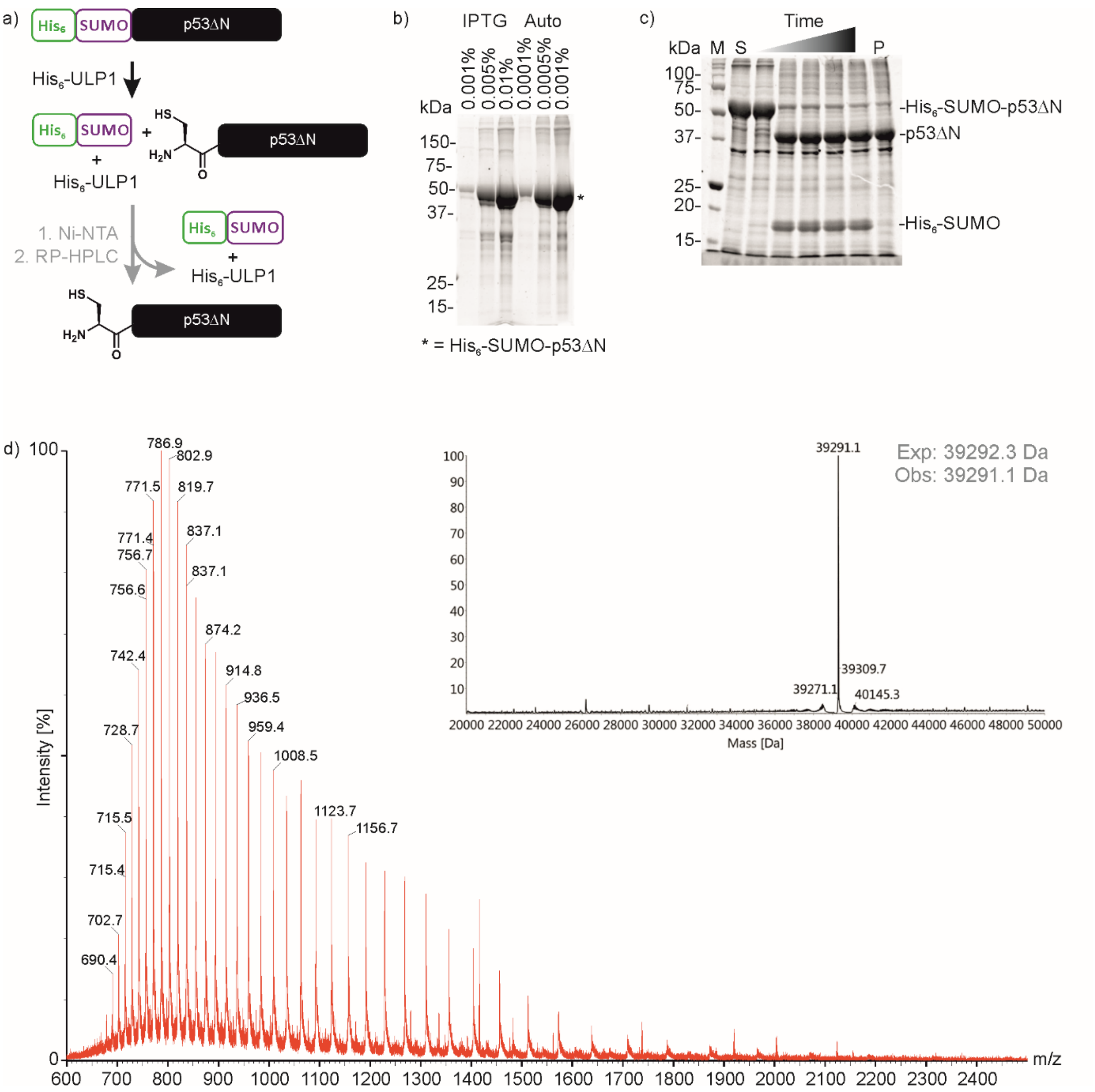

Fig. S1. Production and purification of p53ΔN.

(a) Schematic outline of the production and purification of p53ΔN. (b) SDS-PAGE analysis of His_6-SUMO-p53ΔN production by IPTG- and Autoinduction. Indicated percentages from 1L cultures were analysed after inclusion body preparations. (c) SDS-PAGE analysis of His_6-SUMO tag cleavage. M = Marker lane, S = Starting material, time points correspond to 0,1,2,3 and 4 h, P = p53ΔN after reverse-Ni-NTA purification (prior to RP-HPLC purification). (d) MS of p53ΔN. Inset: Deconvoluted MS of p53ΔN. Panel (d) is an extended version of Fig. 2h. Expected Mass: 39292.3 Da, Observed: 39292.1 Da.

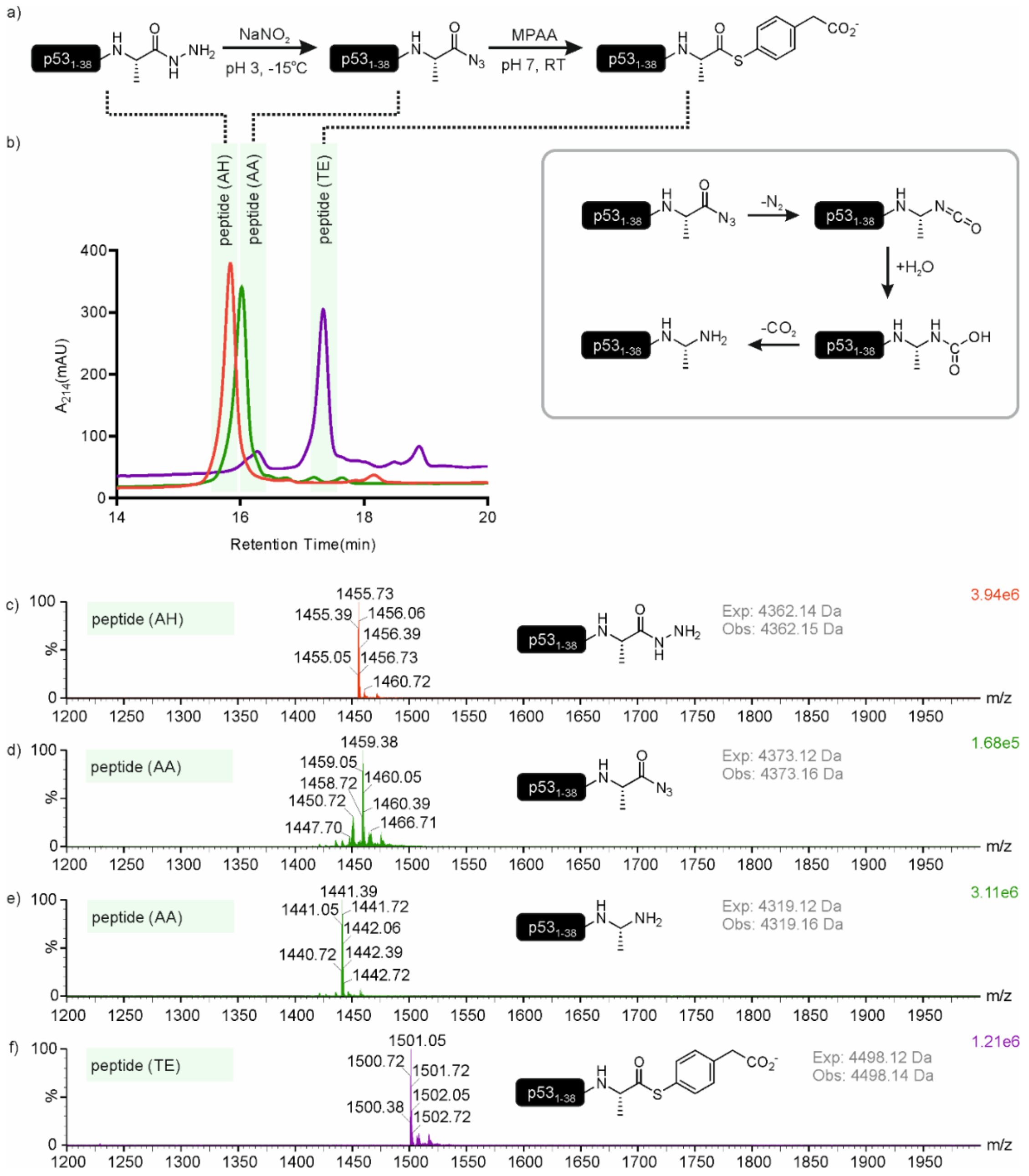

Fig. S2. Optimization of peptide thioesterification.

(a) Schematic outline of the thioesterification reaction. (b) HPLC analysis of the reaction (C18 column, gradient H). (c-f) MS analysis of the reaction intermediates. Intensity scales are indicated on the top right of each MS panel. The main peak in each spectrum has been assigned to the structures indicated. (c) Acyl hydrazide peptide (AH, red): RT=15.8 min, Expected Mass: 4362.14 Da, Observed: 4362.15 Da. (d,e) Acyl azide peptide (AA, green): RT=16.0 min, Expected Mass: 4373.12 Da, Observed: 4373.16 Da. The major species observed by MS analysis is shown in (e) and likely arises from a Curtius degradation during the analysis (see inset; Expected mass: 4319.12 Da, Observed: 4319.16 Da). (f) Peptide MPAA thioester (TE, purple): RT=17.3min, Expected Mass: 4498.12 Da, Observed: 4498.14 Da. We note that this test reaction was carried out with a peptide carrying an N-terminal Met residue. All subsequent reactions were performed with Met1Nle variants.

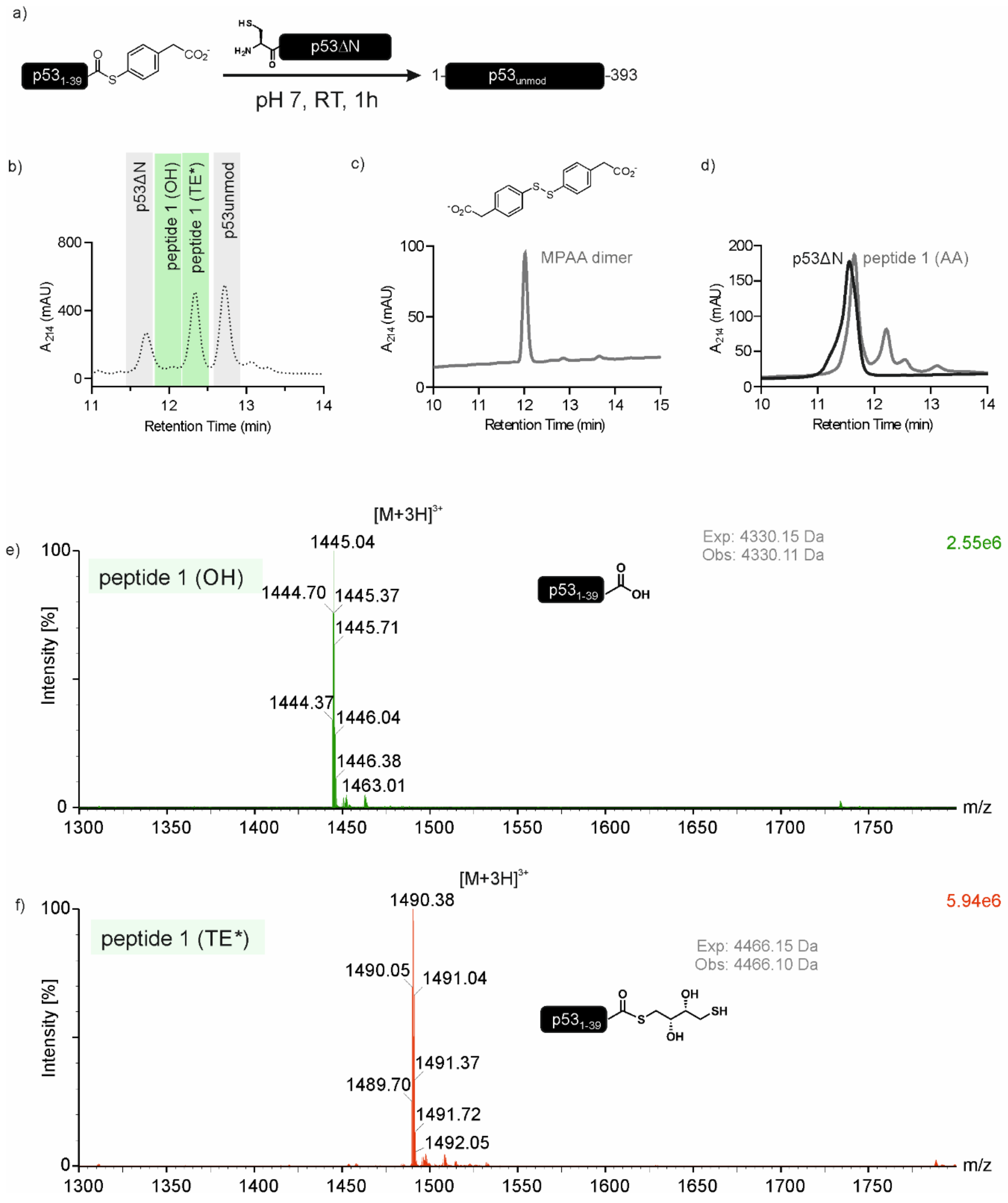

Fig. S3. Identification of reaction intermediates in the ligation of p53$_{unmod}$.
(a) Schematic outline of the ligation reaction to access p53$_{unmod}$. (b) Chromatogram of the ligation reaction taken at 1h (from Fig. 3b; C3 column, gradient F). (c) MPAA dimers co-elute with reaction intermediates, necessitating the addition of 50 mM DTT prior to analysis (C3 column, gradient F). (d) The retention times of peptide **1** acyl azide and p53ΔN partially overlap (C3 column, gradient H). (e,f) MS analysis of HPLC peaks. Notably, the MPAA-thioester (TE) is converted to a DTT-thioester (TE*) when reaction aliquots are reduced with 50 mM DTT prior to analysis.

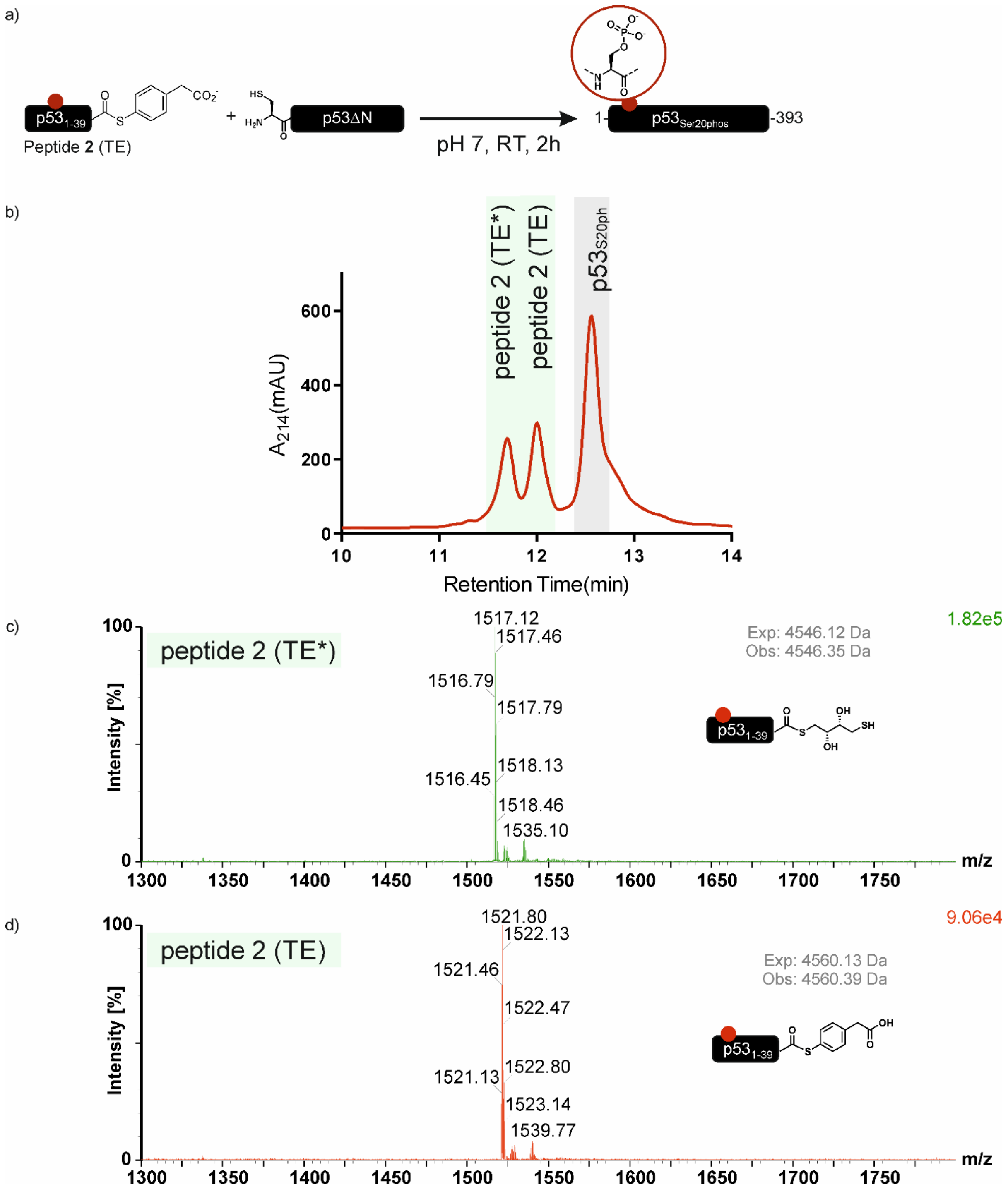

Fig. S4. Identification of reaction intermediates in the ligation of p53$_{S20ph}$.

(a) Schematic outline of the ligation reaction to access p53S20ph. (b) Chromatogram of the ligation reaction taken at 2h (from Fig. 3g; C3 column, gradient F). (c,d) MS analysis of HPLC peaks. Notably, the MPAA-thioester (TE) is partially converted to a DTT-thioester (TE*) when reaction aliquots are reduced with 50 mM DTT prior to analysis.

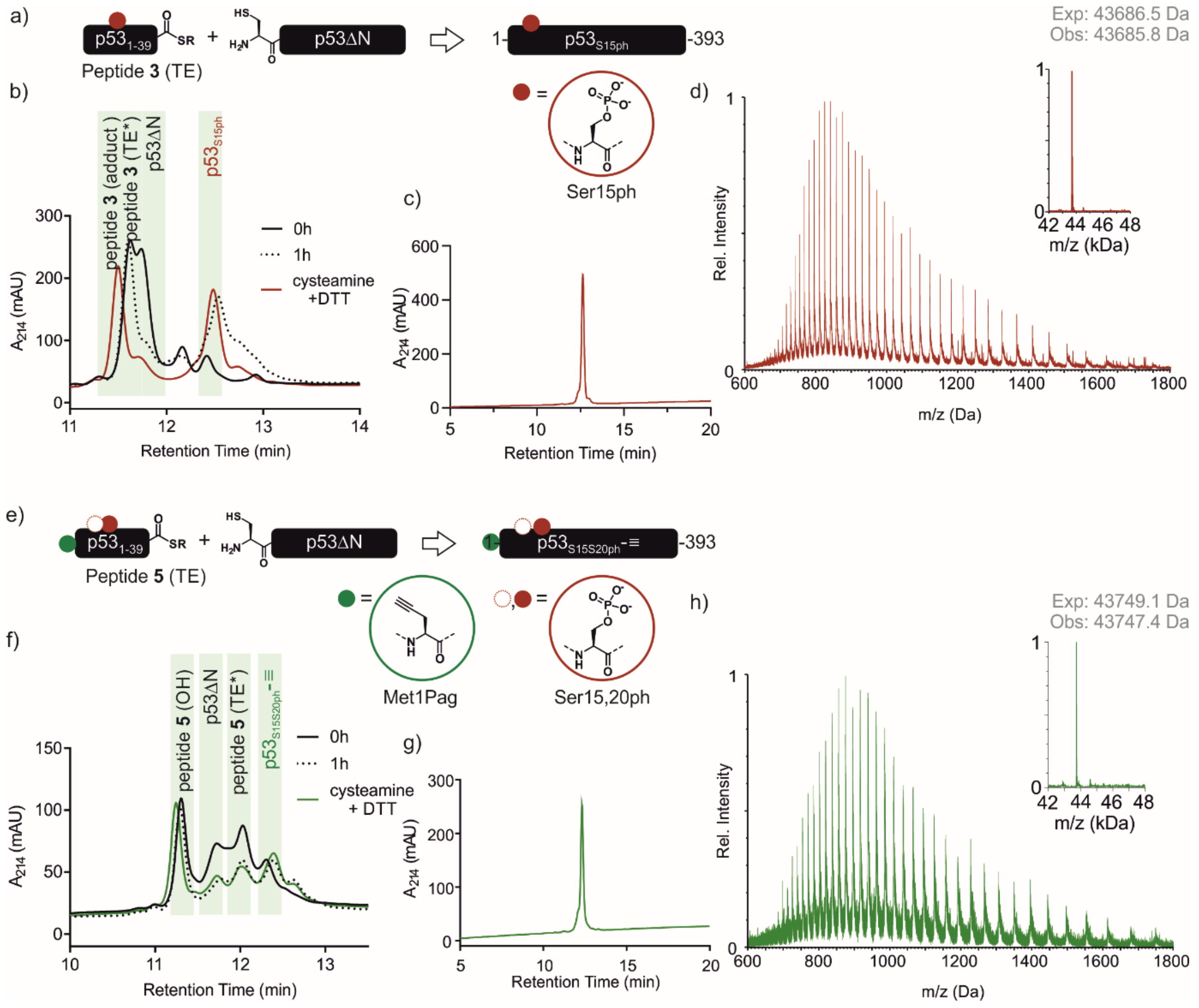

Fig. S5. Native chemical ligation of $p53_{S15ph}$ and $p53_{S15S20ph}$-≡.

a) Schematic representation of the ligation reaction to synthesize $p53_{S15ph}$. b) Time course of the $p53_{S15ph}$ ligation reaction monitored by RP-HPLC. The peptide **3** (adduct) corresponds to the cysteamine adduct after incubation of the peptide **3** TE with cysteamine. c, d) RP-HPLC and MS analysis of purified $p53_{S15ph}$. The deconvoluted spectrum is shown in the inset (expected mass: 43686.5 Da, observed mass: 43685.8 Da). e) Schematic representation of the ligation reaction to synthesize $p53_{S15S20ph\text{-}\equiv}$. f) Time course of the $p53_{S15S20ph\text{-}\equiv}$ ligation reaction monitored by RP-HPLC. g, h) RP-HPLC and MS analysis of purified $p53_{S15S20ph\text{-}\equiv}$. The deconvoluted spectrum is shown in the inset (expected mass: 43749.1 Da, observed mass: 43747.4 Da). Pag = Propargylglycine. The Ser15 and Ser20ph modifications are shown as a red circle and the propargyl residue is shown as a green circle.

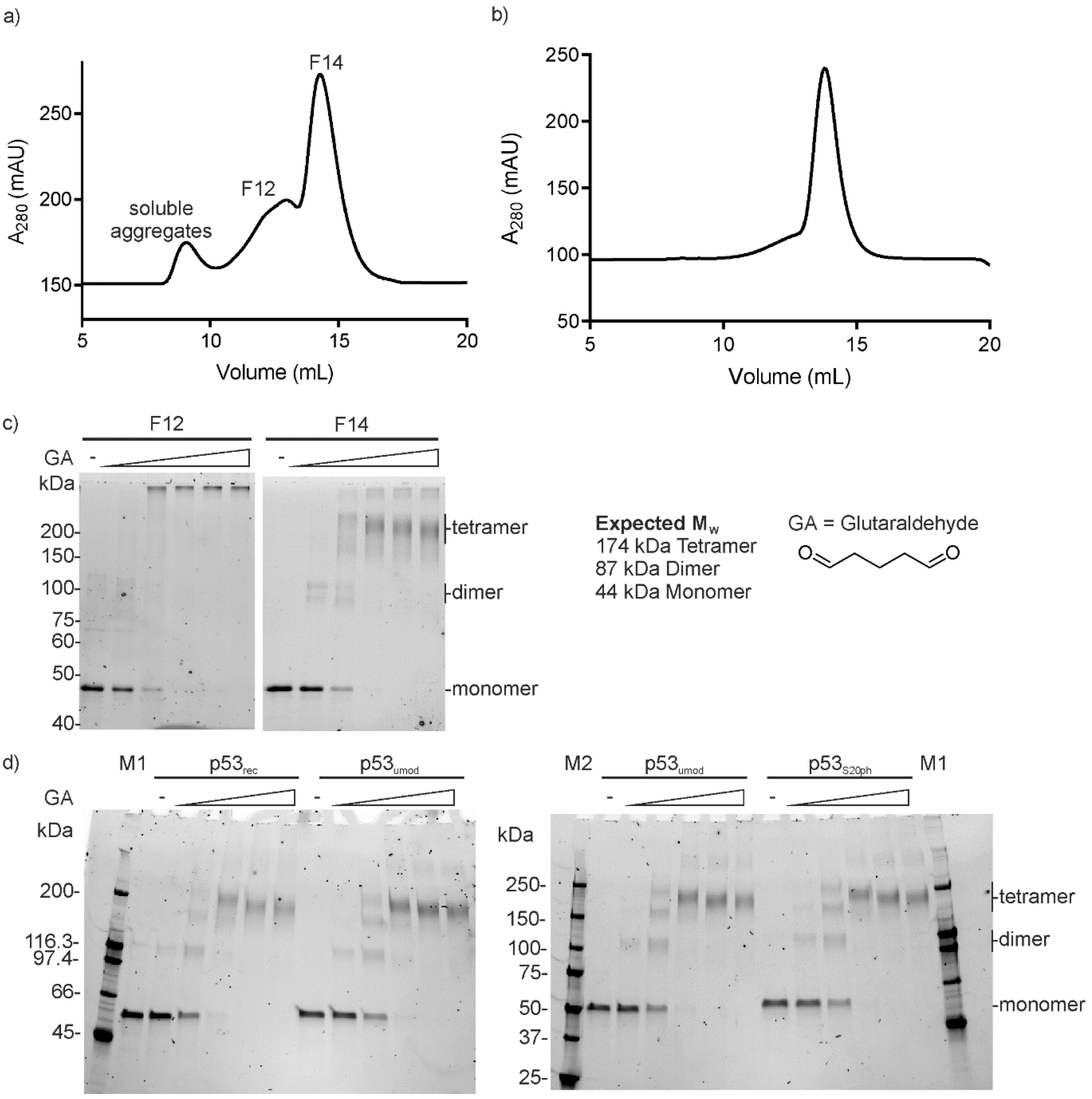

Fig. S6. Analysis of p53 oligomeric state.

(a) Preparative size-exclusion chromatography (SEC) of refolded $p53_{rec}$ on a Superose 6 column. Peaks at 9.1 min, 12-13 min and 14.3 min correspond to soluble aggregates, higher oligomers and tetramers, respectively. (b) Superose 6 SEC of purified p53-F14, re-injected. (c) Glutaraldehyde crosslinking of $p53_{rec}$ size-exclusion fractions. Crosslinking was performed in 0.2 M phosphate buffer for 3 min and quenched by the addition of 1 M Tris, pH 8. Reaction aliquots were denatured by boiling in SDS loading dye and analysed by SDS-PAGE. (d) Crosslinking of the 14 min SEC fraction of p53 variants. Glutaraldehyde concentrations correspond to 0.001-0.1% V/V.

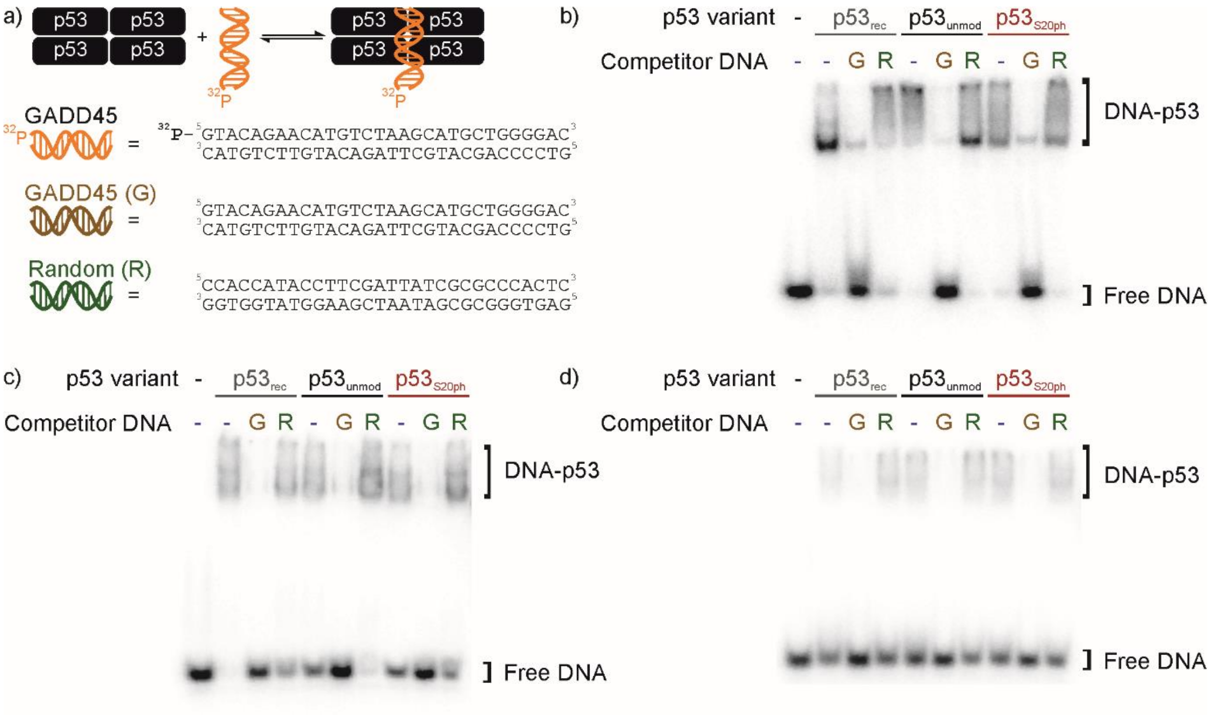

Fig. S7. Refolded p53 binds DNA sequence-specifically.

(a) Schematic outline of DNA-binding assays. Electrophoretic mobility shift assays were performed with 0.4 µM p53 variant (0.1 µM tetramer), 20 nM ^{32}P-labelled DNA and – where applicable – 1 µM unlabelled competitor. DNA sequences for the radiolabelled target DNA (Gadd45, orange), unlabelled Gadd45 competitor (G, brown) and random competitor (R, green) are shown. Three independent replicates are depicted in (b) – (d). Results of the quantification of p53-bound DNA is shown in Fig. 4c.

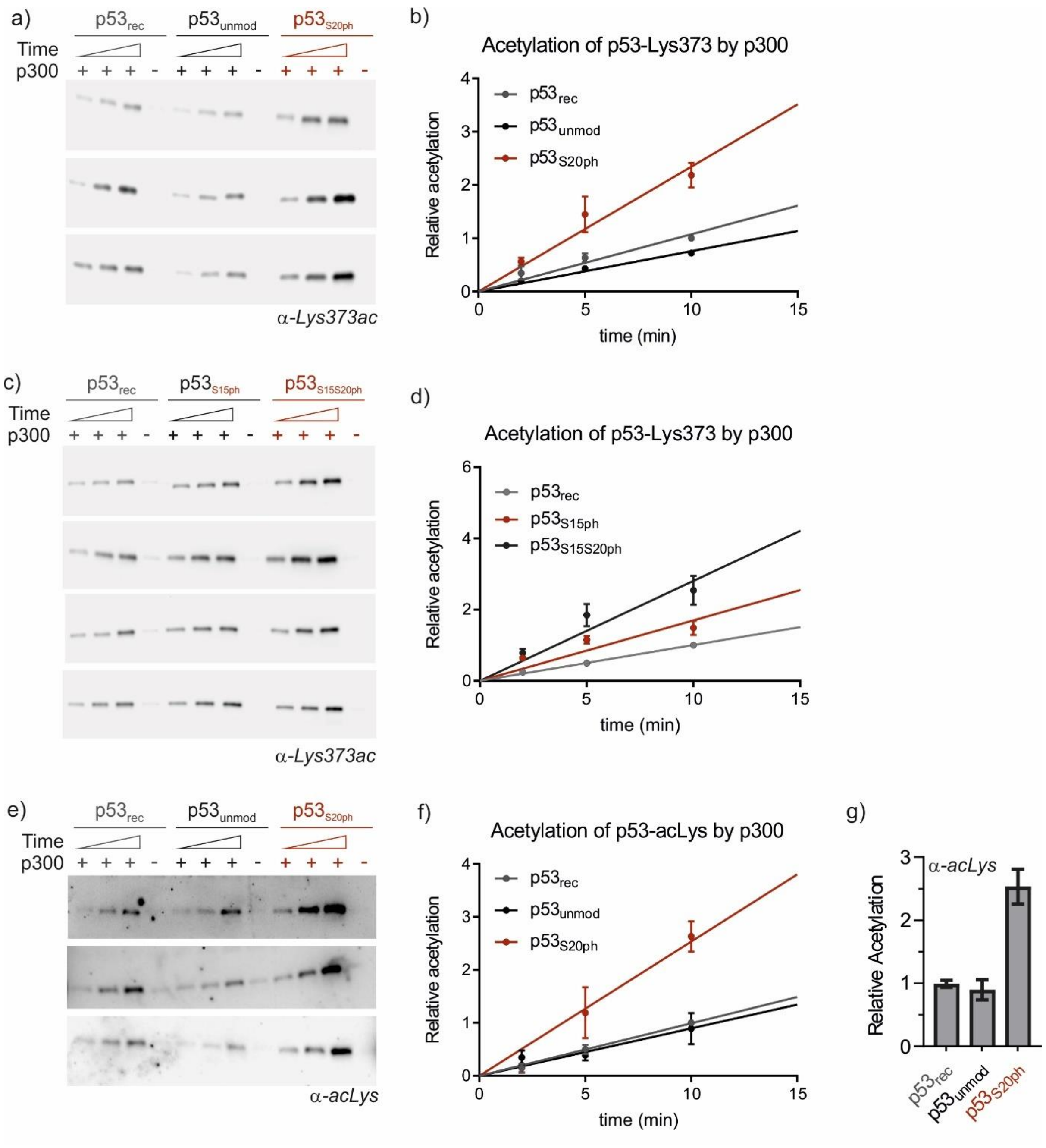

Fig. S8. p53 acetylation assays.

(a-d) Analysis of p300-catalyzed acetylation of p53-Lys373 by western blot. Blots of independent replicates are shown in (a,c) and relative quantifications shown in (b,d). (e,f,g) Analysis of global p53 acetylation catalysed by p300. Blots of 3 independent replicates are shown in (e) and relative quantifications shown in (f). (g) Slopes of the global acetylation curves in (f). Error bars depict the SEM in (b (n=3),d (n=4),f (n=3)) and the error of the fit in (g).

Experimental Procedures

Materials and Methods
Peptide resins, Fmoc-L-amino acids, the monoprotected phosphoserine building block Fmoc-Ser(PO(OBzl)OH)-OH, 2-(7-Aza-1H-Benzotriazole-1-yl)-1,1,3,3-tetramethyluronium hexafluorophosphate (HATU), Oxyma and K-Oxyma were purchased from Novabiochem. Diisopropylethylamine (DIEA), piperidine, phenol Tris(2-carboxyethyl)phosphine hydrochloride (TCEP) were purchased by Merck Sigma-Aldrich and diisopropylcarbodiimide (DIC) from Merck. Peptide synthesis grade Dimethylformamide (DMF) was purchased from Cambridge Reagents Ltd. N-Methyl-2-pyrrolidone (NMP), triisopropylsilane (TIS), thioanisole,1,2-ethanedithiol and Dichloromethane (DCM) from Merck. Peptide grade Trifluoroacetic acid (TFA) was purchased by FluoroChem. Dithiothreitol (DTT) was purchased by AnaSpec.Inc. Reagents and solvents were used without further purification. DNA and native protein concentrations were determined using a NanoDrop ND-8000, 8-Sample spectrophotometer (Labtech). Sequencing of plasmids has been obtained from GENEWIZ. All competent cells were made in-house from commercial strains (TOP10 for cloning, BL21 for expression). Antibodies were used according to manufacturers' instructions, unless otherwise stated. The following antibodies were used:
Primary:
anti-p53: mouse anti-p53 PAb240 (BD Pharmingen, #554166)
anti-p53-pS20: rabbit anti-p53 (phospho S20) antibody (abcam, ab157454)
anti acK373: rabbit anti acK373 (abcam, ab62376)
anti acK: Rabbit Anti-acetyl Lysine antibody (abcam, ab21623)
Secondary:
goat anti rabbit HRP conjugate (Biorad, 170-6515)
goat anti-mouse-HRP conjugate (Biorad 1706516)

General Procedure for Reverse-Phase Chromatography
Analytical and semi-preparative reverse-phase high-performance liquid chromatography (RP-HPLC) was performed on an Agilent 1260 Infinity II instrument equipped with a dual wavelength UV-VIS detector. For analytical work, a 300SB-C3 4.6x150 column and a300SB-C8 4.6x150 were used at a constant flow of 1 mL/min unless stated otherwise. In semi-preparative mode, a 300SB-C3 9.4x250mm column was used at a flow rate of 3.5 mL/min. Typical gradients using mixture of H_2O containing 0.1% TFA (solvent A) and Acetonitrile containing 0.1% TFA (solvent B) are described below.
gradient **A**: 5 min with 25% B, linear gradient from 25-45% B over 40 min; RT
gradient **B**: 5 min with 30% B, linear gradient from 30-40% B over 30 min. RT
gradient **C**: 5 min with 30% B, linear gradient from 30-45% B over 30 min. 50°C
gradient **D**: 2 min with 10% B, linear gradient from 10-70% B over 30 min; RT
gradient **E**: 5 min with 20% B, linear gradient from 25-60% B over 40 min; RT
gradient **F**: 2 min with 20% B, linear gradient from 20-60% B over 20 min; RT
gradient **G**: 5 min with 20% B, linear gradient from 30-55% B over 45 min; RT
gradient **H**: 5 min with 20% B, linear gradient from 20-60% B over 30 min; RT
gradient **I**: 5 min with 20% B, linear gradient from 20-60% B over 30 min; 50°C
HPLC purification of the peptides was performed on an Agilent 1260 Preparative HPLC system using a reversed phase Zorbax 300SB-C3 21.2x150mm 7µm column running at 20 mL/min.

Mass Spectrometry
High-resolution mass spectra (HRMS) were recorded on a Waters Xevo G2-XS QTof after separation by reverse phase chromatography on a Waters Acquity UPLC. The proteins were analysed on a Waters Acquity UPLC Protein BEH C4, 300A, 1.7um, 2.1 x 50mm column, the peptides on a Waters Acquity UPLC BEH C18, 1.7um, 2.1 x 50mm column, with constant flow of 0.2 mL/min using gradient of water containing 0.1% formic acid (solvent C) and acetonitrile containing 0.1% formic acid (solvent D). Typical gradients are described below.
gradient for **proteins**: 2 min with 5% **C**, linear gradient from 5-95% **C** over 4 min; 80°C
gradient for **peptides**: 2 min with 5% **C**, linear gradient from 5-95% **C** over 4.5 min; 60°C

General Procedure for Peptide Synthesis 1
Peptides were synthesized on a Biotage Initiator+ Alstra via Fmoc-based synthesis with DIC/oxyma or DIC/K-Oxyma. Typically, side-chain protected Fmoc-Amino Acids were double coupled at 4 eq. at room temperature for 45 min each. Fmoc-Ser(PO(OBzl)OH)-OH was manually double-coupled with HATU/DIEA, 2 eq. at room temperature for 40 min each. Fmoc deprotection was accomplished with 20% piperidine in DMF twice (one deprotection for 30 sec followed by an additional deprotection for 15 min). Peptides were cleaved from the resin by treatment with reagent K (82.5% TFA, 5% phenol, 5% thioanisole, 5% H_2O, 2.5% 1,2-ethanedithiol) and precipitated with diethyl ether, dissolved in 50% B, lyophilized, and subsequently purified via RP-HPLC with gradient A.

General Procedure for Peptide Synthesis 2
Peptides were synthesized on a Biotage Initiator+ Alstra via Fmoc-based synthesis with DIC/oxyma or DIC/K-Oxyma. Typically, side-chain protected Fmoc-Amino Acids were double coupled at 4 eq. at room temperature for 45 min each. Residues 20-15 including Fmoc-Ser(PO(OBzl)OH)-OH were manually double-coupled with 4 eq. aa/ 4 eq oxyma/ DIEA 0.4 eq/ 4 eq.[1] DIC at room temperature for 60 min each the rest of the sequence residues 1-14 were coupled automatically under the same conditions. Fmoc deprotection was accomplished with 20% piperidine in DMF twice (one deprotection for 30 sec followed by an additional deprotection for 15 min) for residues 21-39 and with 5% piperazine in DMF (one deprotection for 30 sec followed by an additional deprotection for 15 min) for residues 1-20 respectively. Peptides were cleaved from the resin by treatment with reagent K (82.5% TFA, 5% phenol, 5% thioanisole, 5% H_2O, 2.5% 1,2-ethanedithiol) and precipitated with diethyl ether, dissolved in 50% B, lyophilized, and subsequently purified via RP-HPLC with gradient A.

Hydrazine functionalization of resin
The procedure of Stavropoulos et al.[2] was adapted as follows. 286 mg (1 eq, 0.4 mmol) of commercial 2-chlorotrityl chloride resin (substitution: 1.4 mmol/g) were swelled for 15 min in DMF at 4 °C. A mixture of 168 µL (3 eq, 1.2 mmol) of triethylamine and 39.2 µL (2 eq, 0.8 mmol) of hydrazine hydrate in 0.5mL DMF was added dropwise at 4 °C and the suspension was stirred for 60 minutes at room temperature. 2 mL methanol was added in order to quench the excess of reactive chloride sites on the resin. The resin was filtered, washed with DMF (2 x 80 mL), water (2 x 50 mL), DMF (2 x 50 mL), and methanol (2 x 50 mL) then dried under vacuum for 2 h at room temperature.
The C-terminal residue was coupled manually using a mixture of 4 eq of Fmoc-Ala, 4 eq of K-Oxyma and 4 eq of DIC. After 45 min, the resin was filtered and another 4 eq of fresh reagents were added for 45 min. Then, the resin was filtered and washed (Batch-Flow-Flow-Batch with DMF, batch washes for 1 min, flow washes for 30 sec). The rest of the synthesis was carried out on a Biotage Initiator+ Alstra as described above.

Synthesis of p53(1-39)-NHNH$_2$

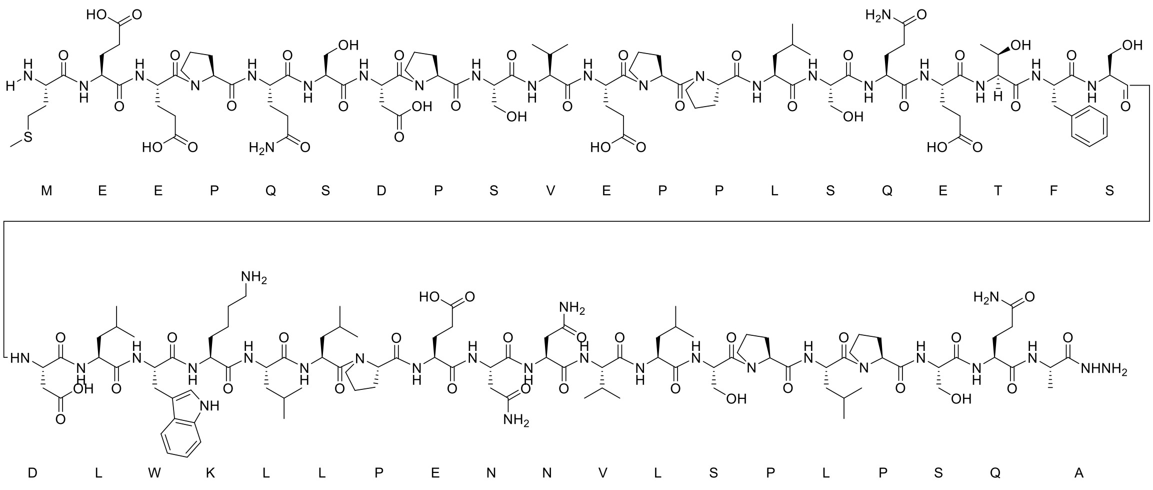

The peptide was synthesized via Fmoc-SPPS as a C-terminal acyl hydrazide as described above. The crude product was purified via preparative RP-HPLC using gradient A on a C18 BS300. Analytical data: RP-HPLC gradient H, RT = 15.8 min (C-18 column); Yield: 6%; Expected Mass: 4362.14 Da, Found: 4362.14 Da. See Supplemental *Fig. S9* for RP-HPLC chromatogram and MS analysis of the purified peptide.

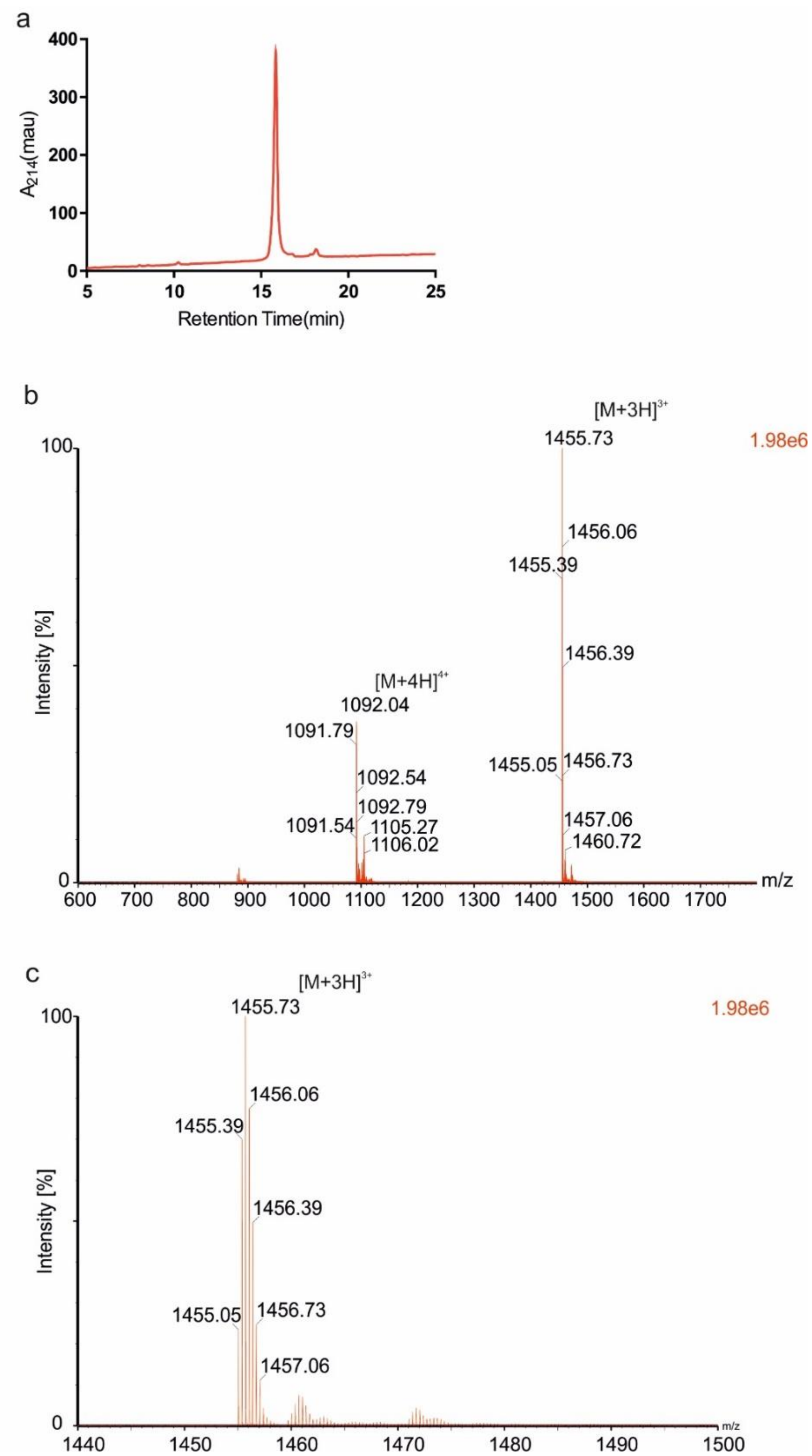

Fig. S9. Analysis of p53(1-39)-$NHNH_2$.

(a) RP-HPLC chromatogram and (b,c) MS analysis.

Synthesis of peptide **1**

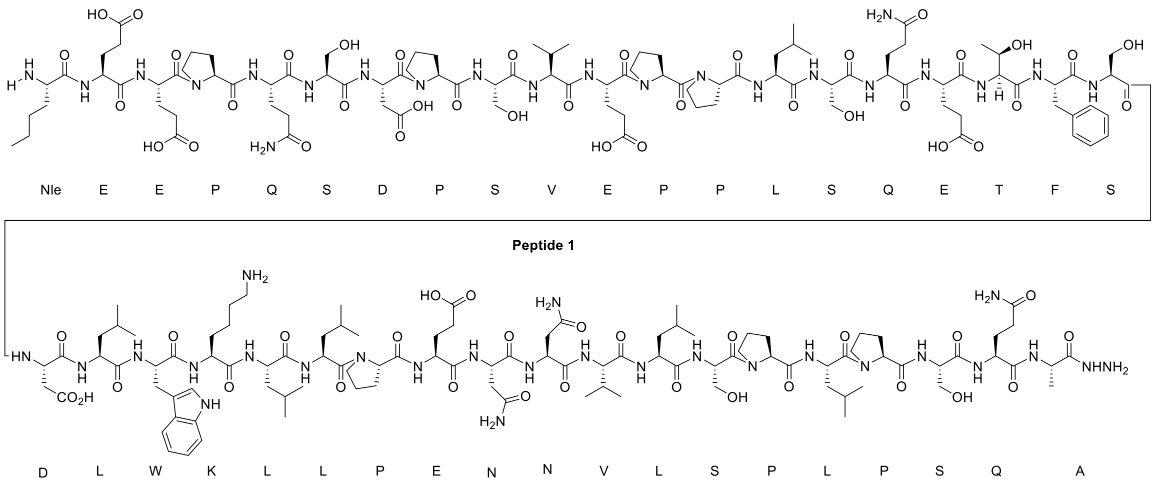

Peptide **1** was synthesized via Fmoc-SPPS as a C-terminal acyl hydrazide as described above. The crude product was purified via preparative RP-HPLC using gradient B. Analytical data: RP-HPLC gradient F, RT = 11.5 min (C-3 column); Yield: 20%; Expected Mass: 4344.18 Da, Found: 4344.24 Da. See Fig. 2a,b and Supplemental Fig. S10 for RP-HPLC chromatogram and MS analysis of the purified peptide.

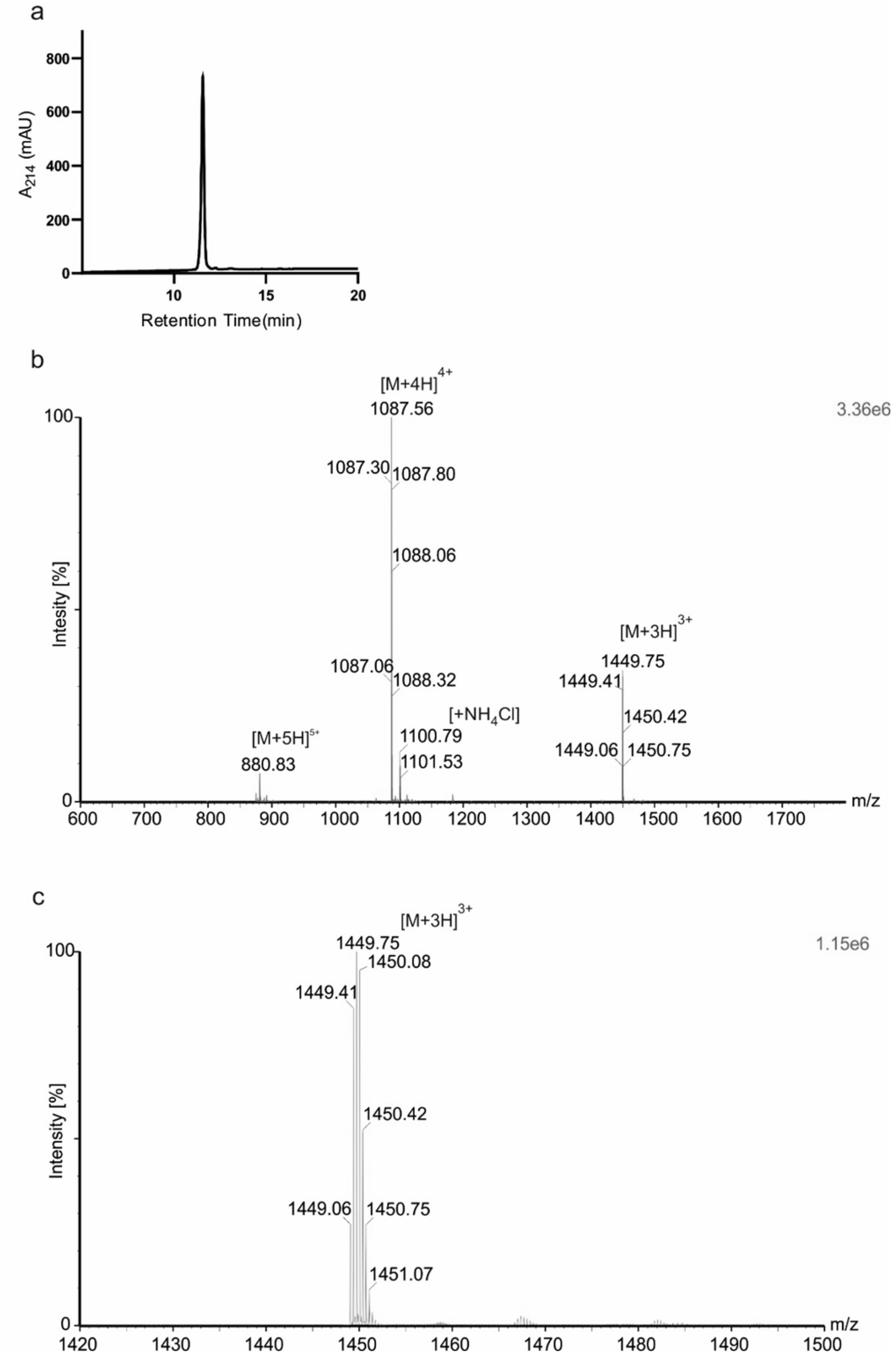

Fig. S10. Analysis of purified peptide 1.

(a) RP-HPLC chromatogram and (b,c) MS analysis.

Synthesis of peptide **2**

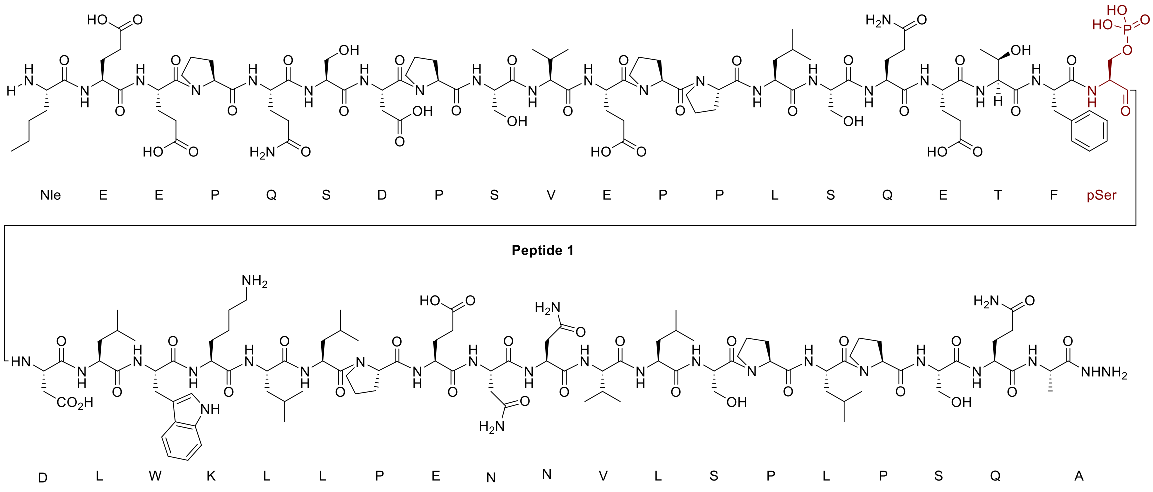

The peptide was synthesized via Fmoc-SPPS as a C-terminal acyl hydrazide as described above. The crude product was purified via preparative RP-HPLC using gradient C. Analytical data: RP-HPLC gradient F, RT = 11.4 min (C-3 column); Yield: 9%; Expected Mass: 4424.15 Da, Found: 4424.16 Da. See Fig. 2e,f and Supplemental Fig. S11 for RP-HPLC chromatogram and MS analysis of the purified peptide.

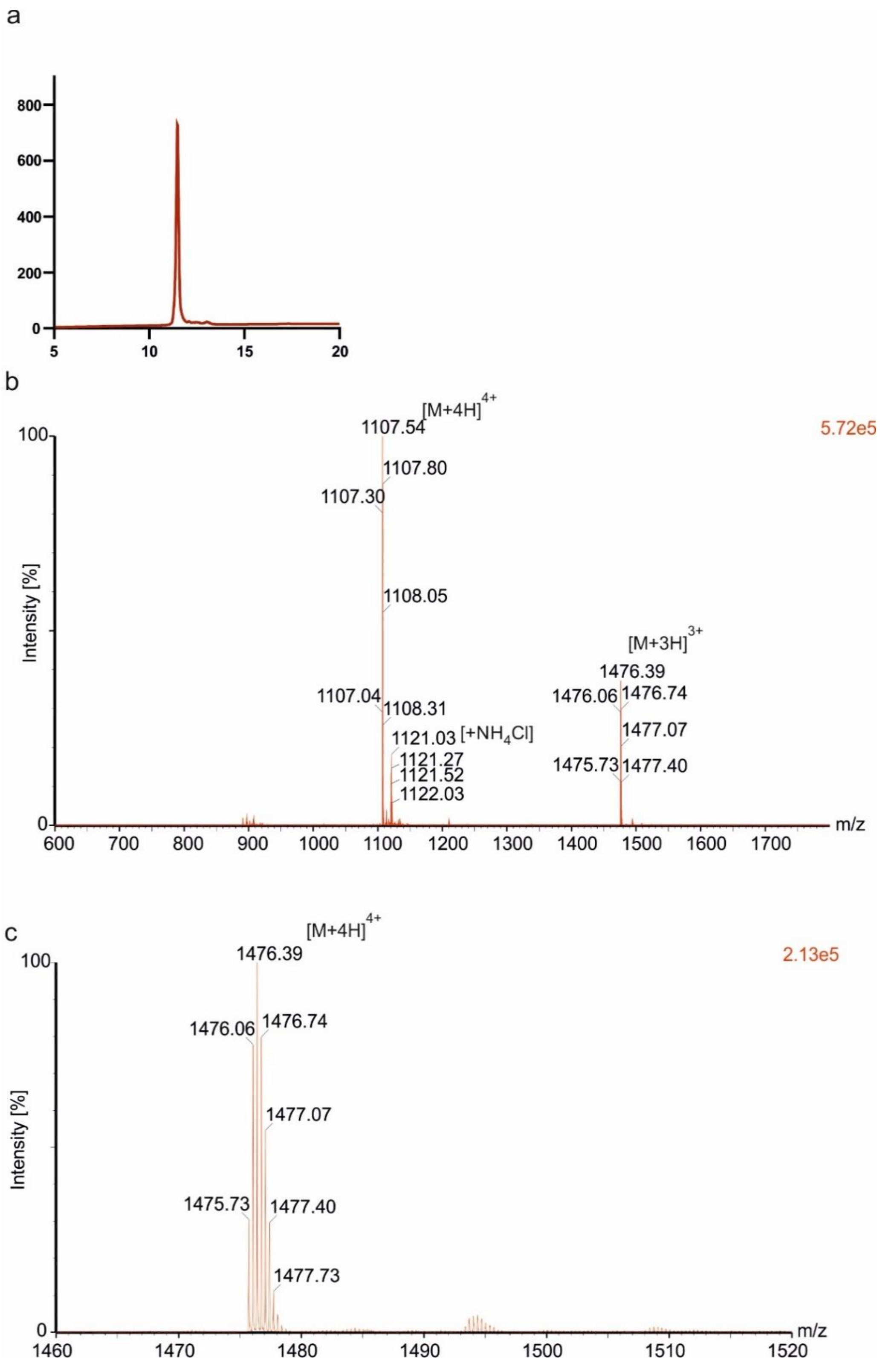

Fig. S11. Analysis of purified peptide 2.

(a) RP-HPLC chromatogram and (b,c) MS analysis.

Synthesis of peptide **3**

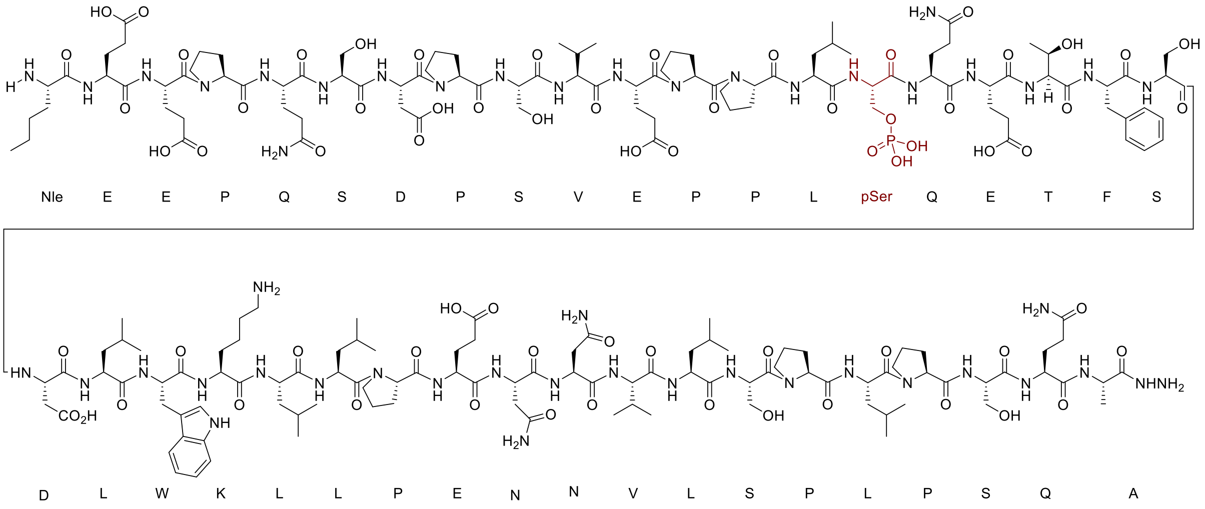

The peptide was synthesized via Fmoc-SPPS as a C-terminal acyl hydrazide as described above according to procedure 2. The crude product was purified via preparative RP-HPLC using gradient C. Analytical data: RP-HPLC gradient F, RT = 11.0 min (C-3 column); Yield: 9%; Expected Mass: 4424.15 Da, Found: 4424.13 Da. See Supplemental Fig. S11 for RP-HPLC chromatogram and MS analysis of the purified peptide.

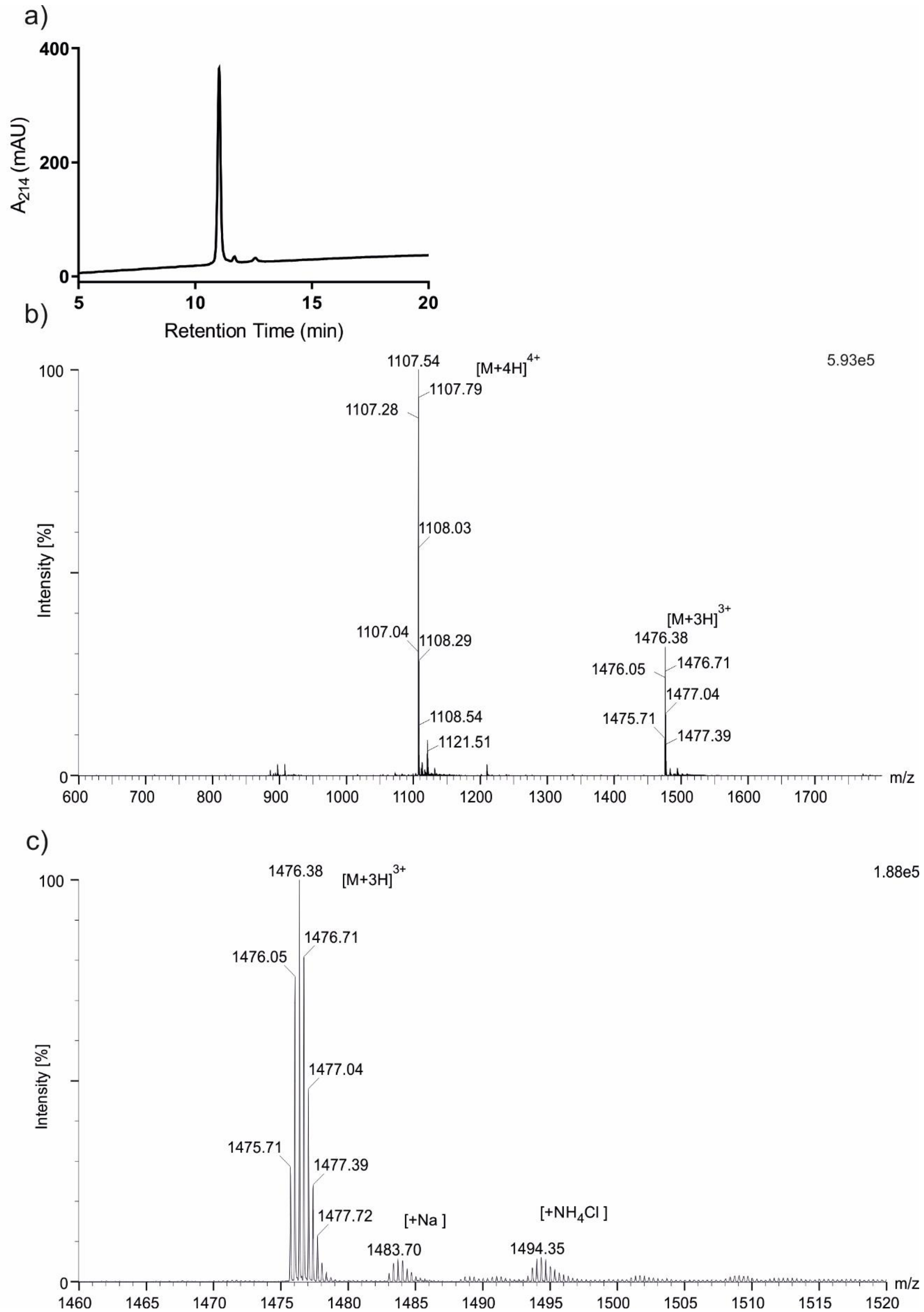

Fig. S12. Analysis of purified peptide 3.

(a) RP-HPLC chromatogram and (b,c) MS analysis.

Synthesis of peptide **4**

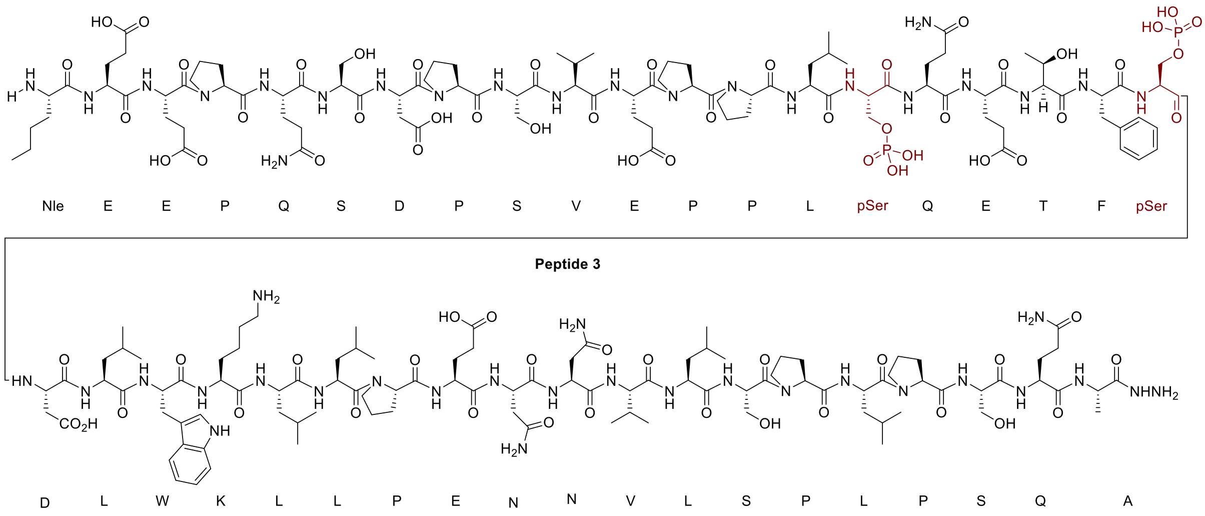

The peptide was synthesized via Fmoc-SPPS as a C-terminal acyl hydrazide as described above according to procedure 2. The crude product was purified via preparative RP-HPLC using gradient C. Analytical data: RP-HPLC gradient F, RT = 10.9 min (C-3 column); Yield: 3.3%; Expected Mass: 4504.11 Da, Found: 4504.14 Da. See Supplemental Fig. S11 for RP-HPLC chromatogram and MS analysis of the purified peptide.

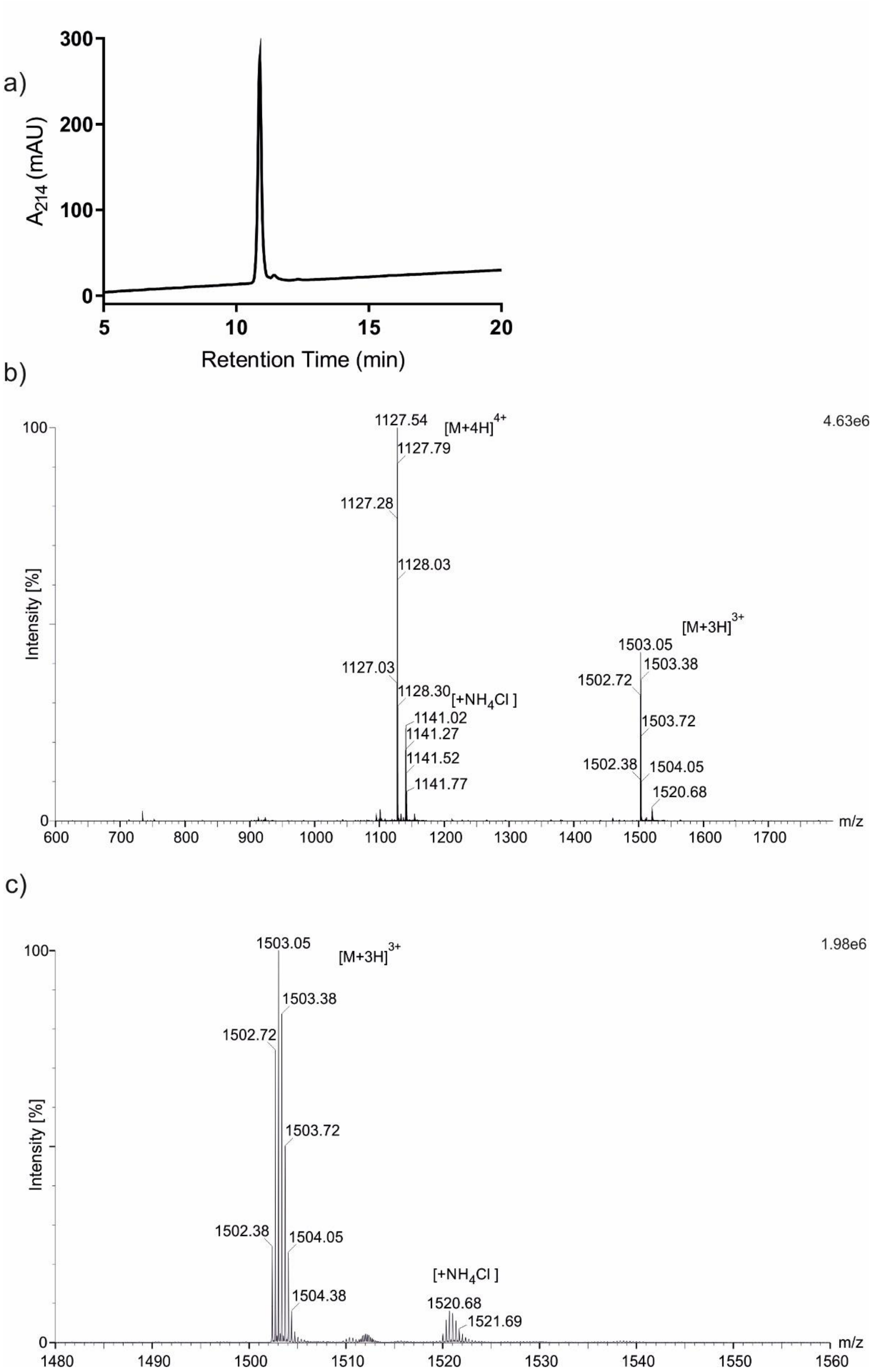

Fig. S13. Analysis of purified peptide 4.
(a) RP-HPLC chromatogram and (b,c) MS analysis.

Synthesis of peptide **5**

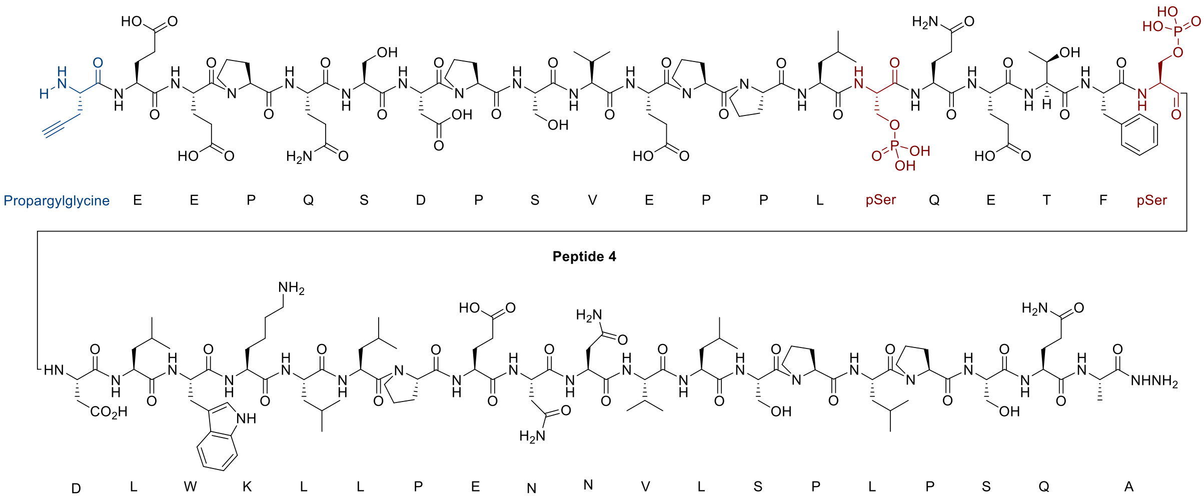

The peptide was synthesized via Fmoc-SPPS as a C-terminal acyl hydrazide as described above according to procedure 2. The crude product was purified via preparative RP-HPLC using gradient C. Analytical data: RP-HPLC gradient A, RT = 19.2 min (C-3 column); Yield: 4.4%; Expected Mass: 4486.07 Da, Found: 4486.08 Da. See Supplemental Fig. S13 for RP-HPLC chromatogram and MS analysis of the purified peptide.

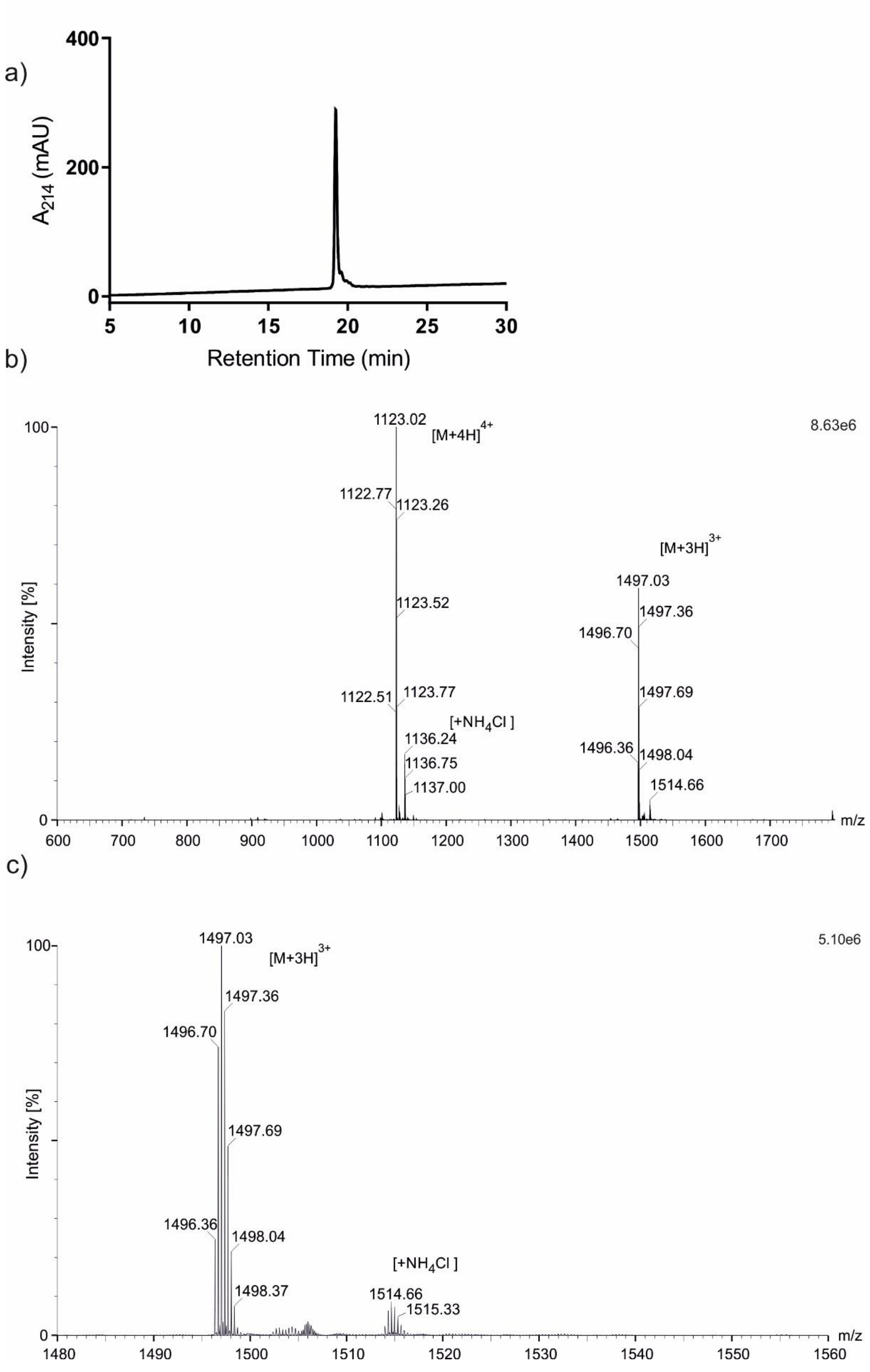

Fig. S14. Analysis of purified peptide 5.

(a) RP-HPLC chromatogram and (b,c) MS analysis.

Synthesis of $p53_{369\text{-}383}$ for EMSAs

The peptide was synthesized via Fmoc-SPPS on a Rink amide resin. The N-terminus was acetylated and a Gly-Tyr sequence was appended to the C-terminus for UV detection (sequence = acLKSKKGQSTSRHKKL-GY-NH_2). Each amino acid was double coupled at 75 °C for 5 min using K-Oxyma and DIC as coupling reagents. Fmoc deprotection was accomplished with 20% piperidine in DMF twice at RT (one deprotection for 3 min followed by an additional deprotection for 10 min). N-terminal capping was done with 50 eq acetic anhydride in DMF and 50 eq DIEA in NMP for 10 min at RT. Peptides were cleaved from the resin by treatment with 95% TFA, 2.5 % TIS, 2.5 % H_2O. The crude product was purified via preparative RP-HPLC using a gradient from 5-30% B. Analytical data: RP-HPLC gradient D, RT = 7.2 min; Yield: 6.5 %; Expected Mass: 1986.15 Da, Found: 1986.17 Da. See Supplemental *Fig. S15* for RP-HPLC chromatogram and MS analysis of the purified peptide.

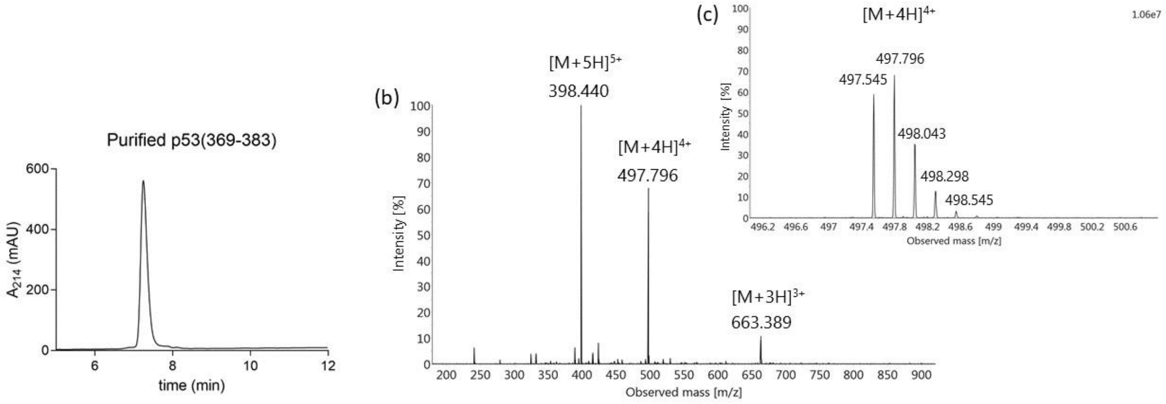

Fig. S15. Analytics of p53369-383 peptide*.*

(a) RP-HPLC trace of purified peptide using gradient D on an analytical C18 RP-HPLC column. (b,c) Mass spectrum (Expected Mass: 1986.15 Da, Found: 1986.17 Da). (c) Isotope distribution of the charge z = +4 peak.

Production and purification of $p53_{rec}$

A plasmid containing p53 (pET3a-p53) was kindly provided by Johannes Buchner [3]. p53 was produced in BL21 via auto-induction [4]. A preculture was grown for 24 h at 37 °C in MDG media and used for 1:1000 inoculation into auto-induction media TYM-5052 (using tryptone instead of N-Z-amine and no trace metals; growth conditions: 1% tryptone, 0.5 % yeast extract, 25 mM Na_2HPO_4, 25 mM KH_2PO_4, 50 mM NH_4Cl, 5 mM Na_2SO_4, 2 mM $MgSO_4$, 0.5 % glycerol, 0.05 % glucose, 0.2 % lactose, 100 µg/mL ampicillin), and grown for 16 h at 37 °C. The cells were harvested by centrifugation (4200 rpm, 30min, 4 °C). Inclusion bodies were isolated as described by Bell *et al.*[3] Pellets were re-dissolved in 0.1 M Tris-HCl, 1 mM EDTA, pH 7 at 4 °C and lysed by incubation for 30 min on ice with 1.5 mg lysozyme/g cells, followed by sonication and passage through a cell disruptor. 10 µg/mL DNase I and 3 mM $MgCl_2$ were added for 30 min at room temperature to remove DNA and the reaction was quenched by the addition of 0.5 volumes of 60 mM EDTA, 6% Triton X-100, 1.5 M NaCl, pH 7. Inclusion bodies were isolated and washed as reported by Bell *et al.*[3] p53 was solubilized from the purified inclusion bodies in 10 mL/L culture IB solubilization Buffer (100 mM Tris–HCl, 6 M Guanidine Hydrochloride (GdmCl), 50 mM DTT, pH 8) by mild resuspension and incubation for 2 h at RT, followed by pH adjustment to 3 using HCl and centrifugation for 30 min at 30,000xg at 4 °C to remove insoluble material. $p53_{rec}$ samples were buffer exchanged into 30% acetonitrile in water containing 0.1% formic acid with PD10 columns and lyophilised for long term storage at -80 °C. Analytical data: RP-HPLC gradient I, RT = 17.8 min (C-18 column); Expected Mass: 43653.2 Da, Found: 43652.1Da. See Supplemental Fig. S16 for purified RP-HPLC chromatogram and MS analysis.

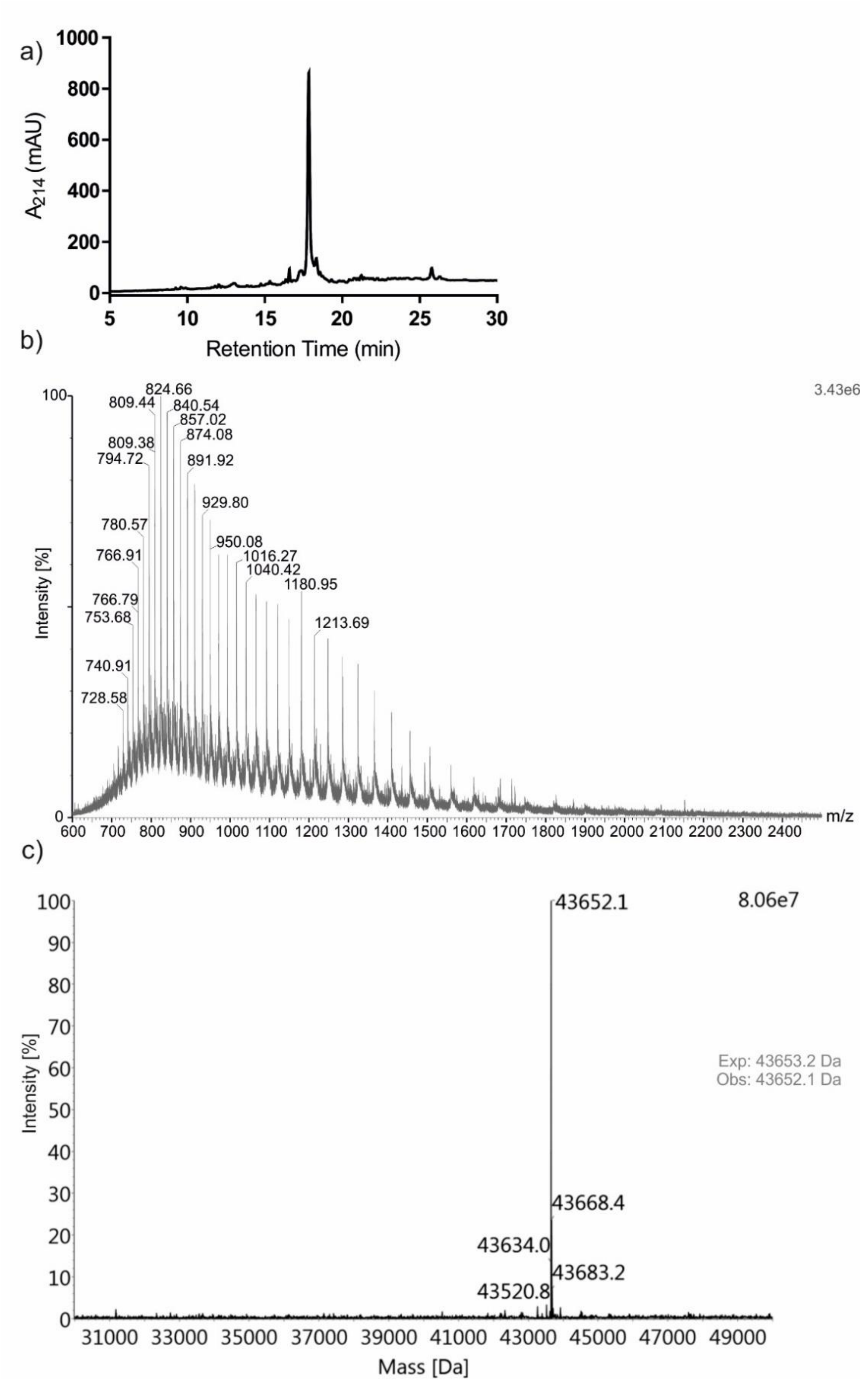

Fig. S16. Analytics of p53$_{rec}$.

(a) RP-HPLC chromatogram, (b,c) MS analysis. Simplified versions of the mass spectrum in (b) and the deconvoluted spectrum in (c).

Construction of His-SUMO-p53ΔN
The Met40Cys mutation was installed into pET15b-p53 (Cheryl Arrowsmith; addgene #24859)[5] by PCR with Q5® High-Fidelity DNA Polymerase (New England Biolabs) using primers GTCCCAAGCAtgcGATGATTTGATGCTGTCCCCG and GGCAAGGGGGACAGAACG (mutation site indicated in lowercase letters). Subsequently, an N-terminal His_6-SUMO tag was appended using the NEBuilder HiFi DNA Assembly Kit (New England Biolabs). The primers used for the His_6-SUMO and vector-p53 fragments were gccatcatcatcatcatcacagcagcggcATGTCGGACTCAGAAGTC + gacagcatcaaatcatcgcaACCACCAATCTGTTCTCTG and GCCGCTGCTGTGATGATG + TGCGATGATTTGATGCTGTCCC, respectively.

The final protein sequence is indicated below (p53ΔN underlined).
MGSSHHHHHHSSGMSDSEVNQEAKPEVKPEVKPETHINLKVSDGSSEIFFKIKKTTPLRRLMEAFAK
RQGKEMDSLRFLYDGIRIQADQTPEDLDMEDNDIIEAHREQIGGCDDLMLSPDDIEQWFTEDPGPDE
APRMPEAAPPVAPAPAAPTPAAPAPAPSWPLSSSVPSQKTYQGSYGFRLGFLHSGTAKSVTCTYSP
ALNKMFCQLAKTCPVQLWVDSTPPPGTRVRAMAIYKQSQHMTEVVRRCPHHERCSDSDGLAPPQH
LIRVEGNLRVEYLDDRNTFRHSVVVPYEPPEVGSDCTTIHYNYMCNSSCMGGMNRRPILTIITLEDSS
GNLLGRNSFEVRVCACPGRDRRTEEENLRKKGEPHHELPPGSTKRALPNNTSSSPQPKKKPLDGEY
FTLQIRGRERFEMFRELNEALELKDAQAGKEPGGSRAHSSHLKSKKGQSTSRHKKLMFKTEGPDSD

Production of p53ΔN
His_6-SUMO-p53ΔN was produced in BL21 via auto-induction and inclusion bodies isolated as described for $p53_{rec}$ above. Test expressions with IPTG induction were conducted with 0.5 mM IPTG (added at OD_{600}=0.6), followed by growth at 37 ºC for 3.5 hr. For tag cleavage, His_6-SUMO-p53ΔN was re-dissolved in IB solubilization buffer (~10 mg/mL) and diluted 1:10 in Dilution buffer (50 mM Tris-HCl, 150 mM NaCl; 4.5 M Urea, pH 7.9). The solution was further diluted with an equal volume of Cleavage buffer (50 mM Tris-HCl, 150 mM NaCl; 6 mM DTT, 300 mM arginine, pH 7.9) followed by the addition of 0.25 volumes of Ulp1 (0.5 mg/mL in 50 mM Tris, 150 mM NaCl, 1 mM DTT, pH 8.2). The final concentration of His_6-SUMO-p53ΔN and Ulp1 are approximately 0.7 mg/mL and 0.02 mg/mL, respectively. The reaction was incubated at room temperature for 1 hour with mixing and monitored by SDS PAGE. After cleavage, the His_6-SUMO fragment and His_6-tagged Ulp1 were removed by passing the solution through a 5 mL HisTrap column. The flow-through containing p53ΔN was reduced with 50 mM DTT at pH 8 for 1 hour. Finally, p53ΔN was purified by preparative RP-HPLC using gradient E and fractions containing pure product were combined and lyophilised. The purity analysis was carried out on analytical HPLC on gradient F and the identity confirmed by MS.

Analytical data: RP-HPLC gradient F, RT = 11.6 min (C-3 column); Yield: 16.8%; Expected Mass: 39292.4 Da, Found: 39291.1Da. See Supplemental Fig. S1 for purified RP-HPLC chromatogram and MS analysis.

General Procedure for Native Chemical Ligation 1
The protocol for Native Chemical Ligation (NCL) was adapted from Liu et al.[6] 2.2 mg (0.5 µmol) of lyophilised peptide acyl hydrazide (encompassing p53 residues 1-39) are dissolved in 200 µL 50 mM phosphate buffer (pH 3.0) containing 6 M GdmCl and 1.5 mM EDTA and stirred for 15 minutes at -15 ºC. To oxidize the peptide hydrazide to the corresponding acyl azide, $NaNO_2$ was added to a final concentration 50 mM. The solution was gently agitated for 15 minutes at -15 ºC. The thioesterification occurred upon addition of 4-mercaptophenylacetic acid (MPAA) to a final concentration of 100 mM. Next, 10 mg (0.25 µmol) of lyophilised p53ΔN were added. The pH was adjusted to 6.9 with 1 M NaOH and the reaction was stirred for 1-2h as indicated in the text. To monitor the reaction by RP-HPLC, 2 µL of the mixture were withdrawn at specified intervals, reduced with 50 mM DTT, diluted to 40 µl with ligation buffer pH 3 and analysed by RP-HPLC with gradient A. To monitor the reaction by SDS-PAGE, 2 µL of the reaction were added to 198 µL 1X SDS-Loading dye. The samples were boiled for 10 mins and 10 µL were loaded on a 11% acrylamide gel. Finally, the reaction was incubated in presence of 50 mM DTT for 1h and purified by semi-preparative RP chromatography using gradient G. Fractions containing pure, full-length p53 were combined and lyophilised.

General Procedure for Native Chemical Ligation 2
The protocol for Native Chemical Ligation (NCL) was adapted from Liu et al.[6] 2.2 mg (0.5 µmol) of lyophilised peptide acyl hydrazide (encompassing p53 residues 1-39) are dissolved in 200 µL 50 mM phosphate buffer (pH 3.0) containing 6 M GdmCl and 1.5 mM EDTA and stirred for 15 minutes at -15 ºC. To oxidize the peptide hydrazide to the corresponding acyl azide, $NaNO_2$ was added to a final

concentration 50 mM. The solution was gently agitated for 15 minutes at -15 ^oC. The thioesterification occurred upon addition of MPAA to a final concentration of 100 mM. Next, 10 mg (0.25 µmol) of lyophilised p53ΔN were added. The pH was adjusted to 6.9 with 1 M NaOH and the reaction was stirred for 1-2h as indicated in the text. To monitor the reaction by RP-HPLC, 2 µL of the mixture were withdrawn at specified intervals, reduced with 50 mM DTT, diluted to 40 µl with ligation buffer pH 3 and analysed by RP-HPLC with gradient A. Finally, the reaction was incubated in presence of 400 mM cysteamine for 1h followed by an incubation with 200 mM DTT and was purified by semi-preparative RP chromatography using gradient G. Fractions containing pure, full-length p53 were combined and lyophilized.

General Procedure for Native Chemical Ligation 3

The protocol for Native Chemical Ligation (NCL) was adapted from Liu et al.[6] 2.2 mg (0.5 µmol) of lyophilised peptide acyl hydrazide (encompassing p53 residues 1-39) and 10 mg (0.25 µmol) of lyophilised p53ΔN are dissolved in 200 µL 50 mM phosphate buffer (pH 3.0) containing 6 M GdmCl and 1.5 mM EDTA and stirred for 15 minutes at -15 ^oC. To oxidize the peptide hydrazide to the corresponding acyl azide, $NaNO_2$ was added to a final concentration 50 mM. The solution was gently agitated for 15 minutes at -15 ^oC. The thioesterification occurred upon addition of MPAA to a final concentration of 100 mM. The pH was adjusted to 6.9 with 1 M NaOH and the reaction was stirred for 1-2h as indicated in the text. To monitor the reaction by RP-HPLC, 2 µL of the mixture were withdrawn at specified intervals, reduced with 50 mM DTT, diluted to 40 µl with ligation buffer pH 3 and analysed by RP-HPLC with gradient A. Finally, the reaction was incubated in presence of 400 mM cysteamine for 1h followed by an incubation with 200 mM DTT and was purified by semi-preparative RP chromatography using gradient G. Fractions containing pure, full-length p53 were combined and lyophilized.

Semi-synthesis of $p53_{unmod}$

Following the procedure above, 2eq of peptide **1** were ligated to p53ΔN. After 2 h, the reaction was quenched with 50 mM DTT and $p53_{umod}$ was purified on a semi-preparative 300SB C3 column using gradient G. Analytical data: RP-HPLC gradient F(C-3 column), RT = 12.6 min; Yield: 26%; Expected Mass: 43606.5 Da, Observed: 43604.4 Da. See Supplemental Fig. S17 for RP-HPLC chromatogram and MS analysis of the purified protein.

The procedure was repeated at a 2x higher scale (1 µmol of peptide **1** and 0.5 µmol p53ΔN). After 2 h ligation the reaction was quenched as described above and $p53_{umod}$ was purified. Analytical data: RP-HPLC gradient F, RT = 12.6 min (C-3 column); Yield: 20%; Expected Mass: 43606.5 Da, Observed: 43605.3 Da. See Supplemental Fig. S18 for RP-HPLC chromatogram and MS analysis of the purified protein.

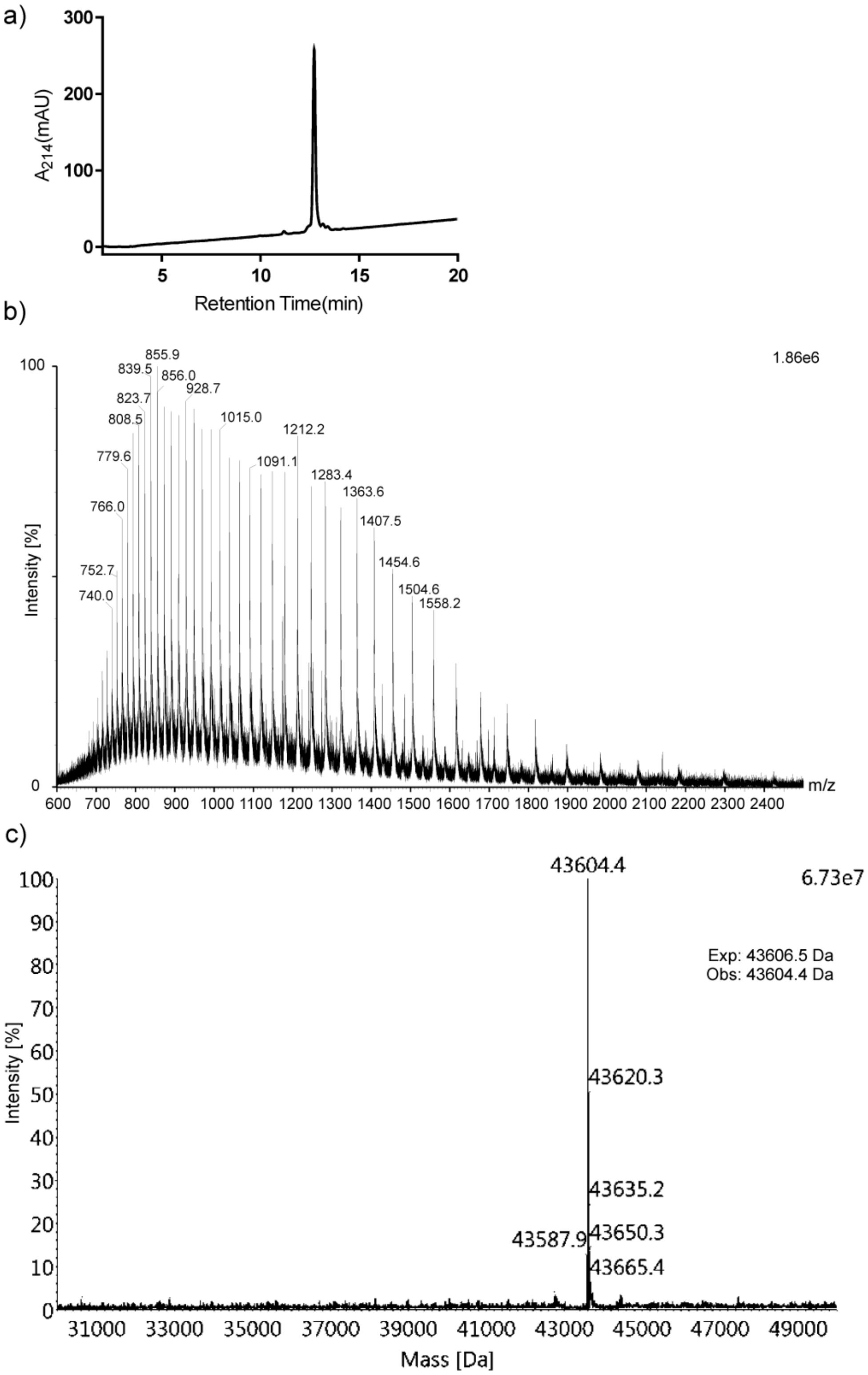

Fig. S17. Analytics of purified p53$_{unmod}$.

The ligation reaction was performed on a scale of 0.25 µmol p53ΔN. (a) RP-HPLC chromatogram, (b,c) MS analysis. Simplified versions of the mass spectrum in (b) and the deconvoluted spectrum in (c) are also shown in Fig. 3d.

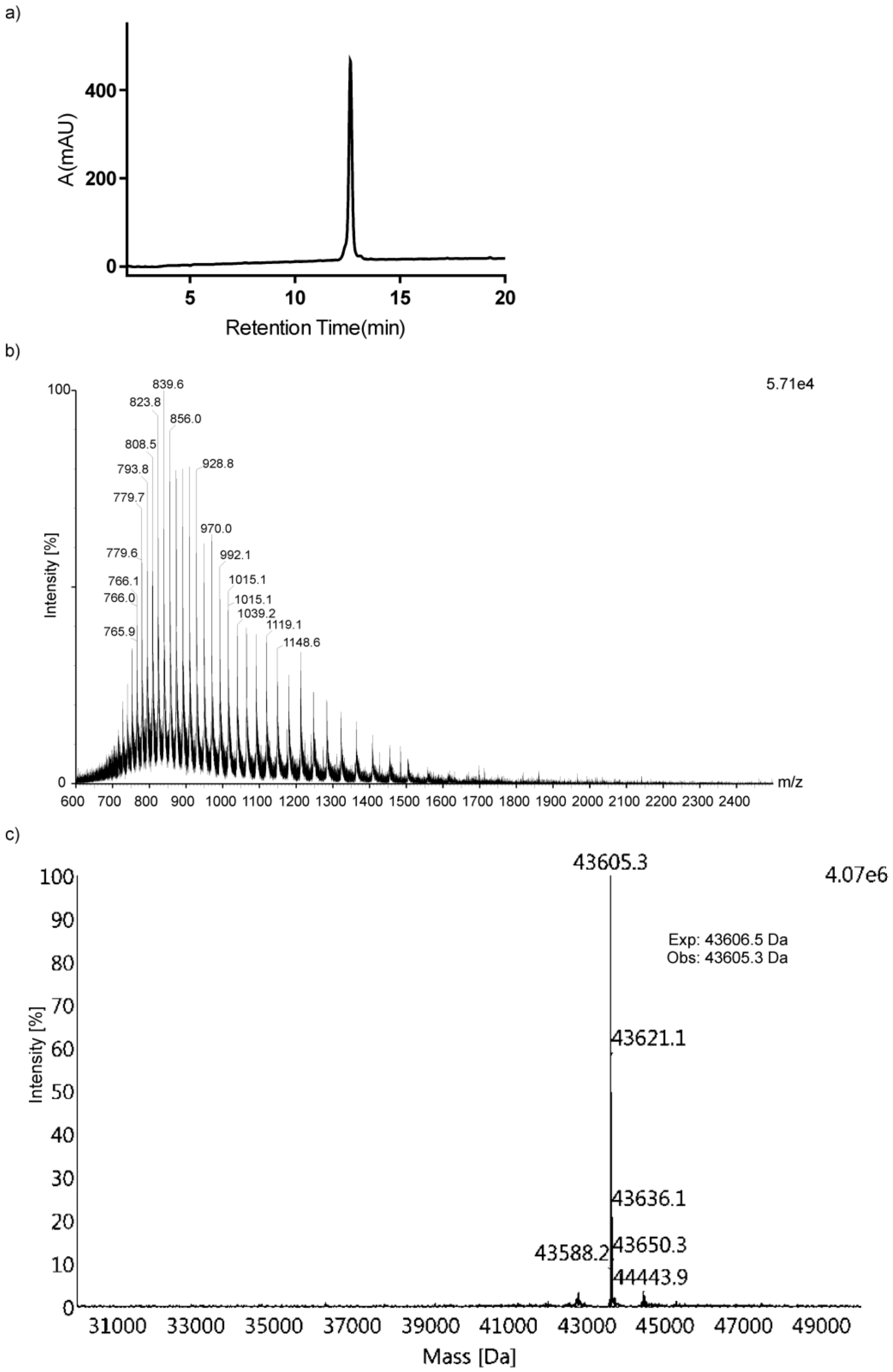

Fig. S18. Analytics of purified p53$_{unmod}$.

The ligation reaction was performed on a scale of 0.5 µmol p53ΔN. (a) RP-HPLC chromatogram, (b,c) MS analysis.

Semi-synthesis of $p53_{S20ph}$

Following the procedure above, 2eq of peptide **2** were ligated to 0.25 µmoles p53ΔN. After 2 h, the reaction was quenched with DTT and $p53_{S20ph}$ was purified. Analytical data: RP-HPLC RT = 12.6 min (C-3 column, gradient F); Yield: 21.6%; Expected Mass: 43687.2 Da, Found: 43686.2 Da. See Supplemental Fig. S19 for purified RP-HPLC chromatogram and Mass Spec analysis.

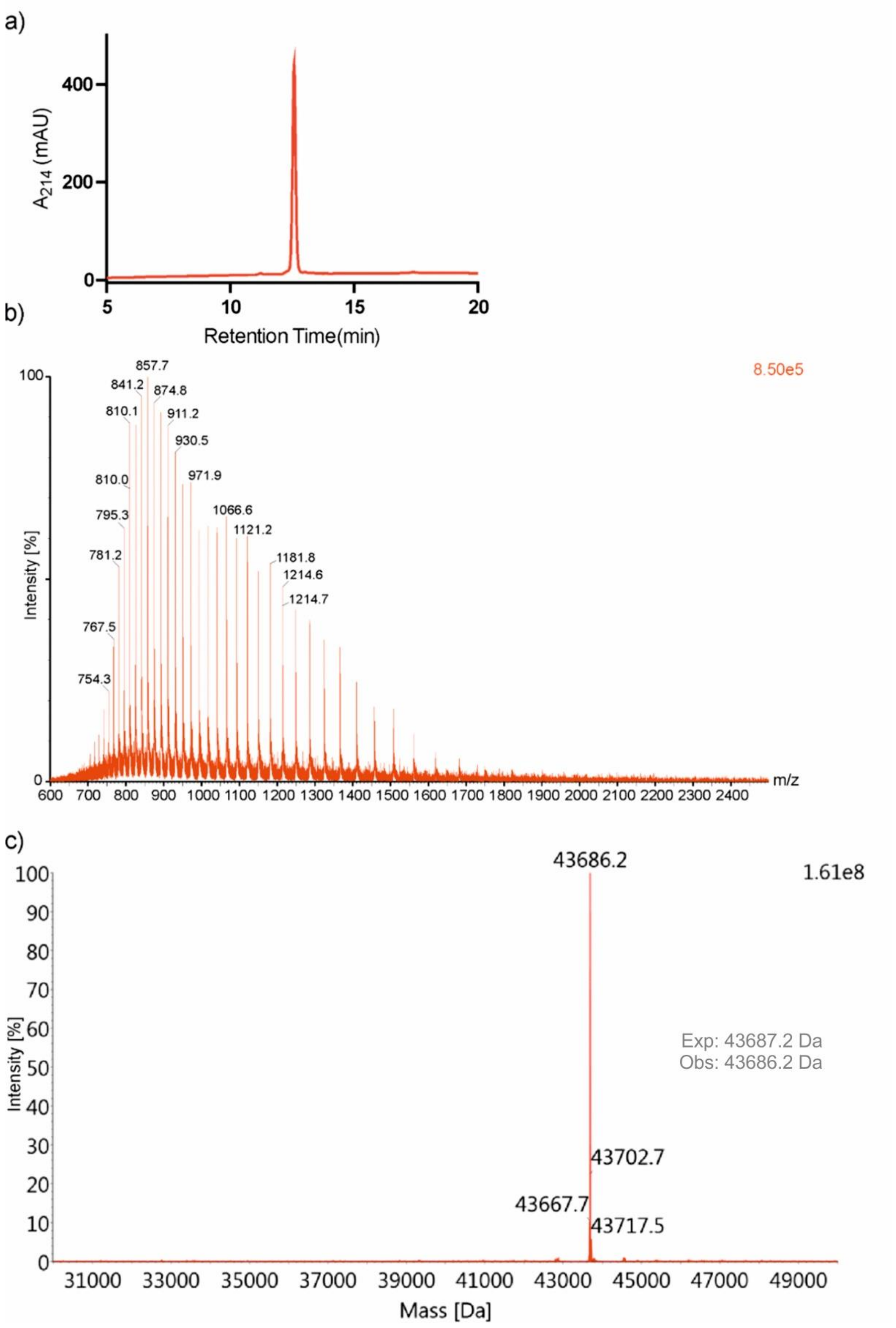

Fig. S19. Analytics of purified p53$_{S20ph}$.

The ligation reaction was performed on a scale of 0.25 µmol p53ΔN. (a) RP-HPLC chromatogram, (b,c) MS analysis. Simplified versions of the mass spectrum in (b) and the deconvoluted spectrum in (c) are also shown in Fig. 3h.

Semi-synthesis of $p53_{S15ph}$

Following the ligation procedure 2, 2eq of peptide **3** were ligated to 0.5 µmoles p53ΔN. After 1 h, the reaction was quenched with DTT and $p53_{S20ph}$ was purified. Analytical data: RP-HPLC RT = 12.6 min (C-3 column, gradient F); Yield: 13.3%; Expected Mass: 43686.5 Da, Found: 43685.8 Da. See Supplemental Fig. S20 for purified RP-HPLC chromatogram and Mass Spec analysis.

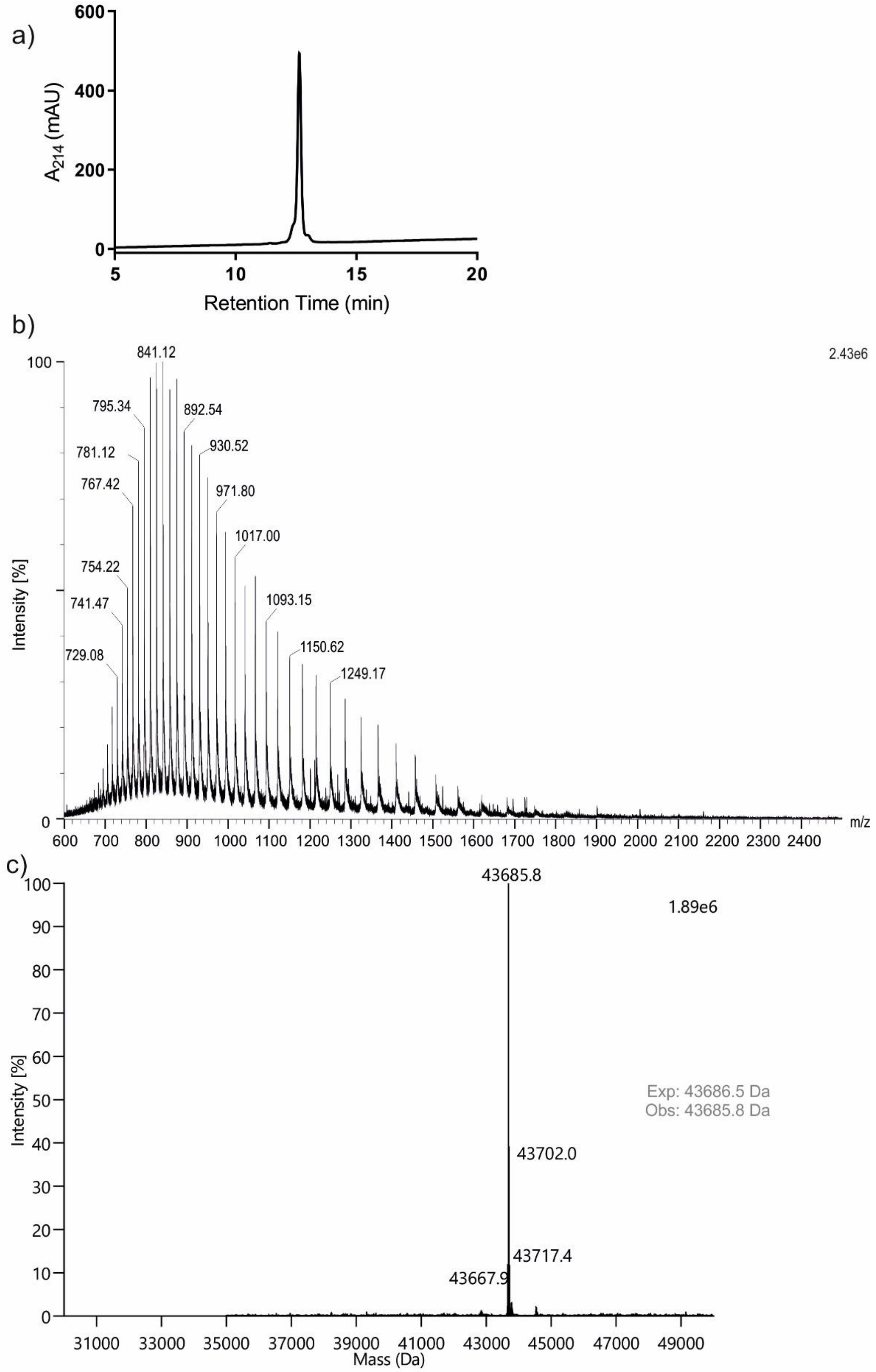

Fig. S20. Analytics of purified $p53_{S15ph}$.

The ligation reaction was performed on a scale of 0.5 µmol p53ΔN. (a) RP-HPLC chromatogram, (b,c) MS analysis. Simplified versions of the mass spectrum in (b) and the deconvoluted spectrum in (c) are also shown in Fig. S5.

Semi-synthesis of $p53_{S15S20ph}$

Following the ligation procedure 2, 2eq of peptide **4** were ligated to 0.5 µmoles p53ΔN. After 1 h, the reaction was quenched with DTT and $p53_{S1520ph}$ was purified. Analytical data: RP-HPLC RT = 12.4 min (C-3 column, gradient F); Yield: 28.1%; Expected Mass: 43767.15 Da, Found: 43765.4 Da. See Supplemental Fig. S19 for purified RP-HPLC chromatogram and Mass Spec analysis.

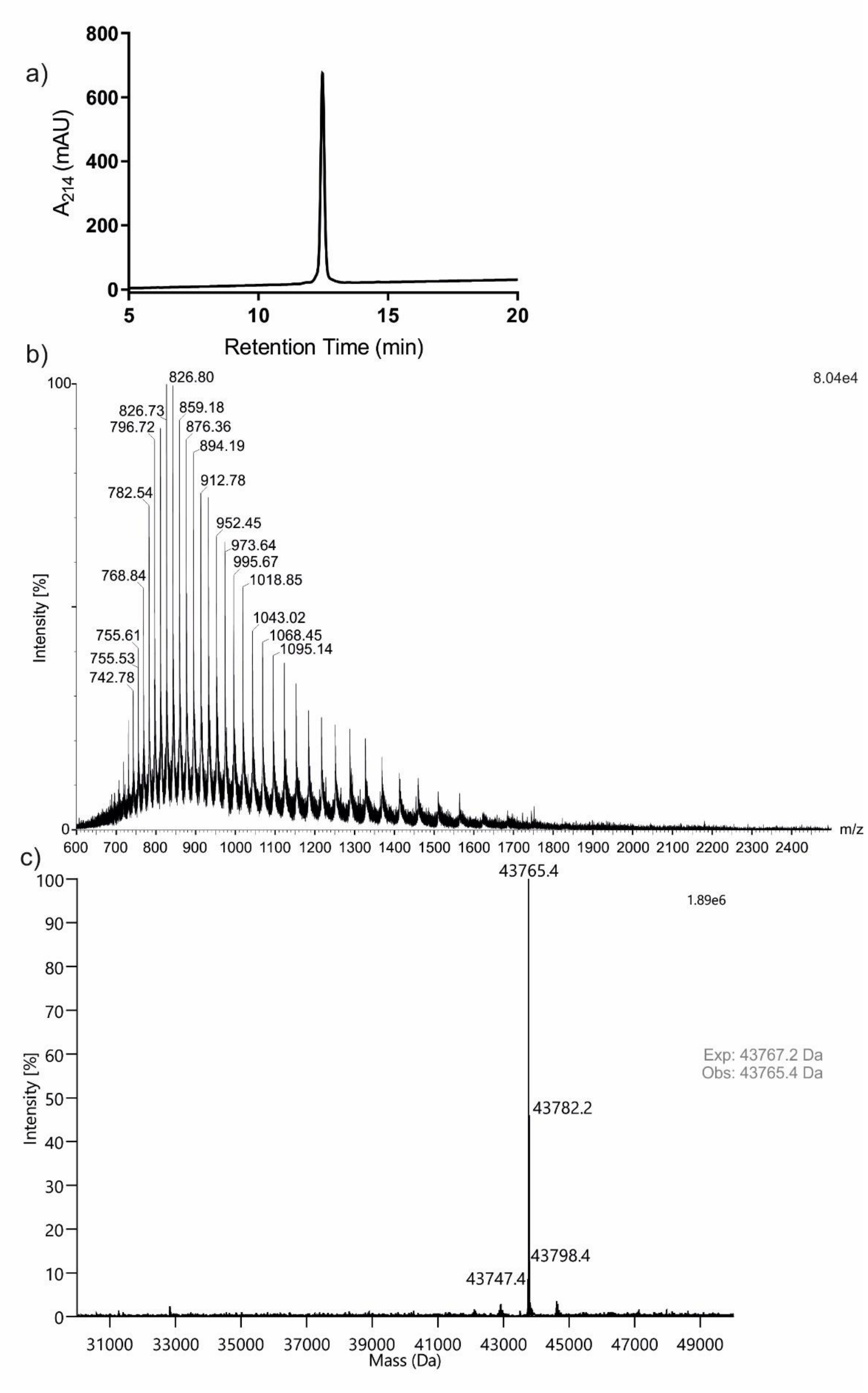

Fig. S21. Analytics of purified p53$_{S15S20ph}$.

The ligation reaction was performed on a scale of 0.5 µmol p53ΔN. (a) RP-HPLC chromatogram, (b,c) MS analysis. Simplified versions of the mass spectrum in (b) and the deconvoluted spectrum in (c) are also shown in Fig. S5.

Semi-synthesis of p53$_{S15S20ph}$-≡

Following the ligation procedure 3, 2eq of peptide **5** were ligated to 0.25 µmoles p53ΔN. After 1 h, the reaction was quenched with DTT and p53$_{S15S20ph}$-≡ was purified. Analytical data: RP-HPLC RT = 12.3 min (C-3 column, gradient F); Yield: 15.5%; Expected Mass: 43749.09 Da, Found: 43747.4 Da. See Supplemental Fig. S20 for purified RP-HPLC chromatogram and Mass Spec analysis.

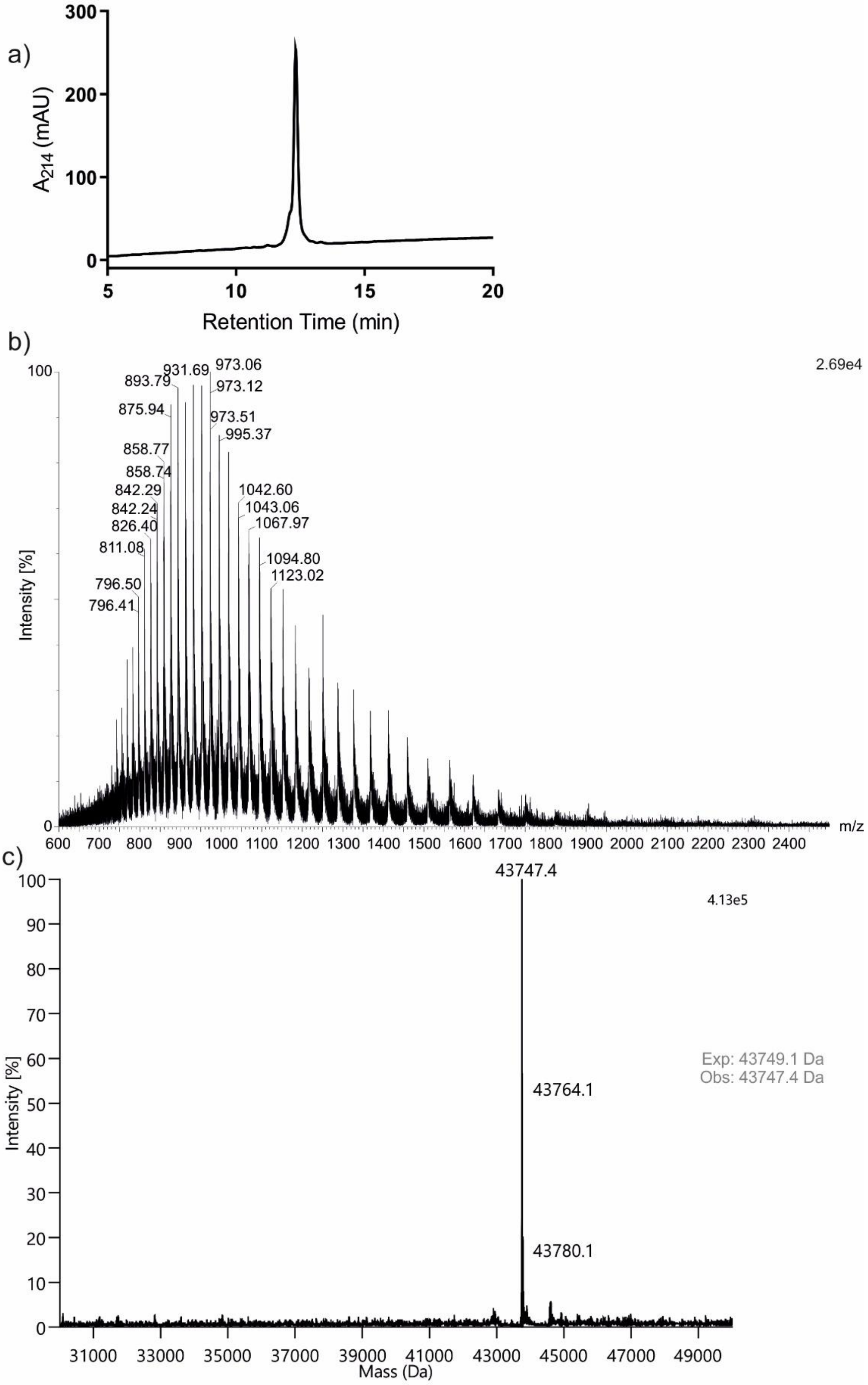

Fig. S22. Analytics of purified p53$_{S15S20ph}$-≡.

The ligation reaction was performed on a scale of 0.25 µmol p53ΔN. (a) RP-HPLC chromatogram, (b,c) MS analysis. Simplified versions of the mass spectrum in (g) and the deconvoluted spectrum in (h) are also shown in Fig. S5.

Refolding of p53

p53 variants were refolded using a modified version of the protocol described by Bell et al.[3] The protein was dissolved in 0.5 mL IB solubilisation buffer to a concentration of ~7 mg/mL. In our hands, determination of p53 concentration by HPLC using ε_{214} = 796501 $M^{-1}cm^{-1}$ calculated based on Kuipers et al.[7] provided the most reliable results. 5 µM stocks of p53 variants were refolded by 1:100 dilution into refolding buffer (50 mM sodium pyrophosphate, 1 M L-arginine, 0.2 mM $ZnCl_2$, 2 mM DTT, pH 8.0) at 15 °C (Total volume: 300ml). A total of six additions, 90 mins apart, were performed, yielding a final concentration of 300 nM p53. After the dilution, the refolded p53 was dialysed twice against 5 L of 50 mM sodium pyrophosphate, 5 % glycerol, 4 mM DTT pH 8.0) at 4 °C (overnight and 4 hr) and centrifuged for 1 h at 4 ^{o}C with 30,000g. The supernatant was loaded onto a 5 mL HiTrap Heparin HP column (GE Healthcare) for 20 h by circulating with a peristaltic pump (flow rate ~ 3 mL/min). The Heparin column was pre-equilibrated in IEX buffer containing 100 mM KCl (30 mM sodium pyrophosphate, 100 mM KCl, 3% (v/v) glycerol, 4 mM DTT, pH 7.5). The protein was eluted by increasing the ionic strength stepwise at a flow rate of ~ 1.5 mL/min (IEX buffer containing: 100 mM KCl (1 CV), 200 mM KCl (1.5 CV), 600 mM KCl (4 CV), 1 M KCl (1.5 CV)). Protein-containing fractions (determined by Bradford assay), were pooled and concentrated by precipitation with 50% ammonium sulphate. The pellets were redissolved in 1/5th of the original volume and loaded onto a Superose 6 Increase 10/300 GL (GE Healthcare) size exclusion column. The Superose 6 column was pre-equilibrated in SEC buffer (30 mM $Na_2H_2P_2O_7$, 300 mM KCl, 3% (v/v) glycerol, 4 mM DTT, pH 7.5). The purified tetramers, eluting at 14 min, were pooled and concentrated by precipitation with 50% ammonium sulphate. The p53 pellets were redissolved in 1/5th of the original volume of SEC buffer and dialysed against p53 storage buffer (30 mM sodium pyrophosphate, 50 mM KCl, 5 % glycerol, 2 mM DTT, pH 7.5), flash frozen in aliquots and stored at -80 ^{o}C. The concentration of refolded p53 was determined by UV spectroscopy using ε_{280} = 35410 $M^{-1}cm^{-1}$.

Characterization of Oligomeric State

Chemical crosslinking. Crosslinking with glutaraldehyde was used to analyse the oligomeric state of refolded p53 fractions. 1.5 µM purified renatured variants in p53 storage buffer were incubated with increasing amounts of glutaraldehyde (stocks prepared in water with final concentrations from 0 to 0.1 % V/V) for 3 min at 37 ^{o}C (reaction volume: 25 µl). The cross-linking reaction was quenched by addition of excess Tris–HCl and the samples were analyzed by SDS PAGE on a 4-15% gradient mini-gel (Bio-rad) or a 7.5 % home-made gel. Expected molecular weights are 44 kDa, 87 kDa and 175 kDa for monomer, dimer and tetramer, respectively.

Analytical size exclusion chromatography. The purity of the tetramers was further investigated by analytical size exclusion chromatography on an Agilent Infinity II HPLC equipped with an AdvanceBio SEC 300A 4.6x300mm, 2.7µm column. All runs were carried out on 30 mM sodium pyrophosphate, 200 mM L-arginine, 300 mM KCl, 3% glycerol, 4 mM DTT, pH 7.5 at room temperature. All species were analysed by injecting 8 µL of p53 tetramer (14 µM stock solution) and detected by absorbance at 280 nm. p53$_{rec}$, p53$_{unmod}$ and p53$_{S20ph}$ were > 90% pure.

DNA Binding Assays

Sequence-specific DNA binding was measured by electrophoretic mobility shift assay as described by Jayaraman & Prives[8] with the modifications of Hupp et al.[9] p53 variants (0.4 µM) were incubated for 20' at RT with 10 µM of a peptide corresponding to the C-terminus of p53$_{369-383}$ and 2.5 µg/mL pUC19 as competitor DNA in a total of 9 µL EMSA buffer (20 mM HEPES pH 7.9 at RT, 1 mg/mL BSA, 50 mM KCl, 5 mM $MgCl_2$, 5 mM DTT, 0.5 mM EDTA, 20 % glycerol, 0.05 % Triton). Subsequently, 1 µL ^{32}P-radiolabelled DNA (double stranded DNA with the sequence GTACAGAACATGTCTAAGCATGCTGGGGAC) was added to a final DNA concentration of 20 nM, and the reaction incubated for 20' at RT. 4 µL of each reaction were separated on a native, pre-run 4% acrylamide gel containing 0.1% Triton-X100 in 0.25x TBE for 25' at 180 V at 4 °C. The gel was soaked with a solution of 4% glycerol, 20% ethanol in water and dried for 2 h at 80 ^{o}C under reduced pressure. Radiolabelled DNA was visualized by phosphorimaging on a BAS IP MS 2025 screen (approximately 1.5 h developing time) with a Typhoon FLA 7000 imaging system (GE Healthcare).

Competition experiments were done using 1 µM unlabelled GADD45 double stranded DNA or 1 µM randomized double stranded DNA with the sequence CCACCATACCTTCGATTATCGCGCCCACTC. The p53-bound fraction of DNA was calculated using ImageJ by dividing the intensity of the shifted DNA population by the sum of intensities of shifted and free DNA. Gels from 3 independent replicates as well as averages and SEM of the quantifications are shown in Fig. S7.

Acetyltransferase assays

Human p300, recombinant, N-terminal FLAG-Tag (Active Motif #81158) was diluted to 0.1 mg/mL with 25 mM HEPES-NaOH pH 7.5, 300 mM NaCl, 10% glycerol, 0.04% Triton X-100 and 0.5 mM TCEP, flash frozen in 2 µL aliquots and stored at -80 °C until use.

p53 (0.3 µM) was incubated with 25 µM acetyl-CoA in acetylation buffer (50 mM Tris pH 8 at 30 °C, 0.5 mM EDTA, 50 mM KCl, 5 mM $MgCl_2$, 10 % glycerol, 1 mM DTT, 0.1 mg/mL BSA) for 5 minutes at 30 °C. p300 (0.15 nM) was added, and acetylation monitored by western blotting reactions after 2, 5, and 10 minutes. A negative control without p300 was analysed after 10 minutes. For analysis, 8 µL of the reaction mixture were quenched by the addition of 2.7 µL 4x SDS loading buffer. The proteins were then separated by SDS-PAGE on a 10% TGX Criterion gel (Biorad; 1x SDS Running Buffer, 47 minutes at 200 V). Proteins were visualized with stain free imaging on a Biorad ChemiDoc MP Imager and subsequently transferred to a PVDF membrane (1.3A, 7 min via the Trans-Blot Turbo system). Membranes were blocked with 3% milk in TBS-T for anti-acK373 blot and 3 % BSA in TBS-T for anti-all-acK blot and probed using either anti-acK373 (abcam, ab62376, rabbit monoclonal, 1:5000 in TBS-T for 1 h at RT) or anti-all-acK (abcam, ab21623, rabbit polyclonal, 1:500 in TBS-T containing 0.1% BSA for 2 h at RT), respectively. The membrane was visualised by chemiluminescence on Biorad ChemiDoc MP Imager after incubation with goat anti-rabbit HRP secondary antibodies (Biorad, 170-6515). The acetylated signals were quantified using ImageJ and the background signal (from an empty lane) subtracted. Relative acetylation was calculated by normalizing signals to the 10 min time point of $p53_{rec}$. No correction for background reaction or antibody cross-reactivity was applied because the signal in the absence of p300 was ≤ 10% in all cases. Relative rates were calculated by linear regression and normalized to the rate of $p53_{rec}$. The error bars represent the standard deviation from three independent assays.

SI References

1. CEM Application Note AP0124 (2018). "*CarboMAX - Enhanced Peptide Coupling at Elevated Temperature.*" Retrieved from CEM website: http://cem.com/media/contenttype/media/literature/AppNote_CarboMAX_ap0124_1.pdf.
2. Stavropoulos, G.; Gatos, D.; Magafa, V.; Barlos, K., Preparation of polymer-bound trityl-hydrazines and their application in the solid phase synthesis of partially protected peptide hydrazides. *Letters in Peptide Science* **1975,** *17* (1964), 1498-1500.
3. Bell, S.; Hansen, S.; Buchner, J., Refolding and structural characterization of the human p53 tumor suppressor protein. *Biophysical Chemistry* **2002,** *96* (2-3), 243-257.
4. Studier, F. W., Protein production by auto-induction in high-density shaking cultures. *Protein Expression and Purification* **2005,** *41* (1), 207-234.
5. Ayed, A.; Mulder, F. A. A.; Yi, G.-S.; Lu, Y.; Kay, L. E.; Arrowsmith, C. H., Latent and active p53 are identical in conformation. *Nature Structural Biology* **2001,** *8* (9), 756-760.
6. Zheng, J.-S.; Tang, S.; Qi, Y.-K.; Wang, Z.-P.; Liu, L., Chemical synthesis of proteins using peptide hydrazides as thioester surrogates. *Nature Protocols* **2013,** *8* (12), 2483-2495.
7. Kuipers, B. J. H.; Gruppen, H., Prediction of Molar Extinction Coefficients of Proteins and Peptides Using UV Absorption of the Constituent Amino Acids at 214 nm To Enable Quantitative Reverse Phase High-Performance Liquid Chromatography−Mass Spectrometry Analysis. *Journal of Agricultural and Food Chemistry* **2007,** *55* (14), 5445-5451.
8. Jayaraman, L.; Prives, C., Activation of p53 sequence-specific DNA binding by short single strands of DNA requires the p53 C-terminus. *Cell* **1995,** *81* (7), 1021-1029.
9. Hupp, T. R.; Sparks, A.; Lane, D. P., Small peptides activate the latent sequence-specific DNA binding function of p53. *Cell* **1995,** *83* (2), 237-245.
